# Atomistic Origin of Structural, Vibrational, and Electronic Fingerprints in Iminopyridine Iron(II) Dichloride Complexes

**DOI:** 10.1021/acs.inorgchem.6c02696

**Published:** 2026-09-04

**Authors:** Anna Maria Fovanna, Giuseppe Leone, Marco Cavazzini, Laura Falivene, Elena Groppo

**Affiliations:** † Department of Chemistry, NIS Interdepartmental Research Center and INSTM Reference Center, 9314 University of Torino, via G. Quarello 15/A, Torino I-10135, Italy; ‡ CNR-Istituto di Scienze e Tecnologie Chimiche “Giulio Natta” (SCITEC), via A. Corti 12, Milano I-20133, Italy; § CNR-Istituto di Scienze e Tecnologie Chimiche “Giulio Natta” (SCITEC), via Golgi 19, Milano I-20133, Italy; ∥ Department of Chemistry and Biology, 19028 University of Salerno, via Ponte don Melillo, 24, Fisciano, Salerno 84084, Italy

## Abstract

Density functional theory (DFT) investigations were performed on a diverse library of iron­(II) dichloride complexes bearing iminopyridine (ImPy) ligands to establish robust structure–property relationships governing their electronic and vibrational behavior. The pronounced structural flexibility of ImPy ligands generates multiple coordination motifs and spin-state manifolds, which frequently complicate experimental characterization and spectroscopic assignment. By systematically correlating geometric descriptors with infrared (IR) and ultraviolet-visible (UV–Vis) signatures, we identify reliable markers of both spin states and local coordination environments across mononuclear and dinuclear complexes. The computational trends show strong agreement with experimental benchmarks, establishing a diagnostic spectroscopic interpretation framework of coordination geometry and spin states in structurally flexible ImPy–Fe complexes, even in the absence of crystallographic data. Ultimately, this study offers a valuable framework for the informed design of next-generation iron (pre)­catalysts, helping to tune electronic landscapes and spin-state populations for optimized reactivity.

## Graphical abstract

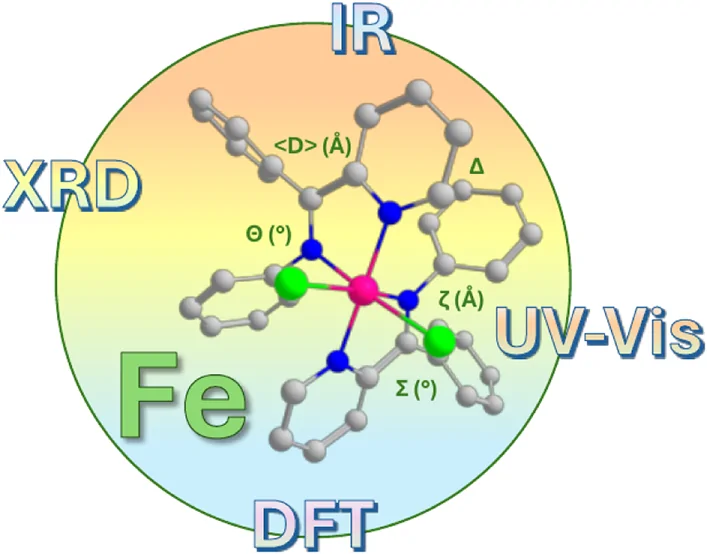

## 1 Introduction

Iron­(II) dichloride complexes bearing iminopyridine (ImPy) ligands have attracted considerable attention owing to their prominent role in homogeneous catalysis and remarkable electronic flexibility, which facilitates the fine-tuning of reactivity.^1–3^ In particular, ImPy–Fe complexes have emerged as efficient alternatives to precious metal catalysts for the synthesis of fine chemicals,^4–7^ and for the polymerization of 1,3-conjugated dienes to synthesize elastomers.^8–20^ Beyond their catalytic utility, ImPy–Fe complexes serve as an ideal platform for exploring structure–property relationships in first-row transition metal chemistry, as their electronic landscape is highly sensitive to subtle modifications in ligand architecture and coordination environment. A hallmark of ImPys is their bidentate N^N chelating motif, which synergically combines significant σ-donor ability with tunable π-acceptor character. This delicate electronic balance enables ImPys to finely modulate the ligand field at the Fe­(II) center, often leading to a narrow energy gap between the high-spin (HS) and low-spin (LS) states.^21–24^ As a consequence, ImPy–Fe complexes exhibit pronounced spin-state variability, making them ideal candidates for exploring how structural distortions, Fe–N bond metrics, and ligand substitution patterns govern vibrational and electronic responses. Understanding these relationships is important not only for interpreting the spectroscopic behavior of ImPy–Fe complexes, but also for guiding the rational design of iron catalysts in which ligand structure and spin state can be tuned to optimize catalytic performance.

Despite the apparent simplicity of their synthesis, which typically involves direct reaction between the target ligand and iron­(II) dihalides, the resulting complexes display a surprising degree of structural diversity. Variations in ligand substitution and ligand-to-metal ratio can give rise to a rich landscape of coordination motifs, spanning from mononuclear tetrahedral and pseudo-octahedral species to dinuclear architectures, and various neutral or ion-paired configurations. Notably, the stoichiometric ratio of the reactants does not necessarily dictate the identity of the complex ultimately isolated in the solid state. Reactions performed with a nominal ImPy:FeCl_2_ = 1:1 ratio may afford: (*i*) monoligated neutral (ImPy)­FeCl_2_ species with tetrahedral geometry at the iron center (Scheme 1a),^9,12,13,25^ (*ii*) bis-ligated (ImPy)_2_FeCl_2_ octahedral complexes (Scheme 1b),^10,26^ (*iii*) chloride-bridged unsymmetrical dinuclear structures of the type L_2_Fe­(*μ*-Cl_2_)­FeCl_2_ (Scheme 1c), featuring two iron centers in distinct coordination environments,^10,15^ and (*iv*) an ion pair consisting of a bis-ligated [(ImPy)_2_FeCl]^+^ cation with a distorted trigonal bipyramid geometry at the iron center and a [FeCl_4_]^−^ anion (Scheme 1d).^11^ Such structural diversity reflects the pronounced conformational adaptability of ImPys, which can accommodate different coordination environments while preserving strong chelation through the N^N donor set. Single-crystal X-ray diffraction (XRD) has been instrumental in revealing this structural richness; however, crystallization of ImPy–Fe complexes is often challenging. Moreover, the specific solvents and parameters required for crystal growth can bias the isolation toward certain motifs, which do not necessarily reflect the dominant species in solution or in the bulk solid. Consequently, while crystallographic data offer valuable structural insights, relying solely on their interpretation may not yield an unambiguous spin-state assignment; such data should ideally be complemented by additional experimental evidence. This issue is exacerbated by the limited availability of high-resolution spectroscopic benchmarks among reported studies on related iron complexes, particularly vibrational and electronic signatures, capable of discriminating between closely related structural configurations or spin-state manifolds.

**Scheme 1:**
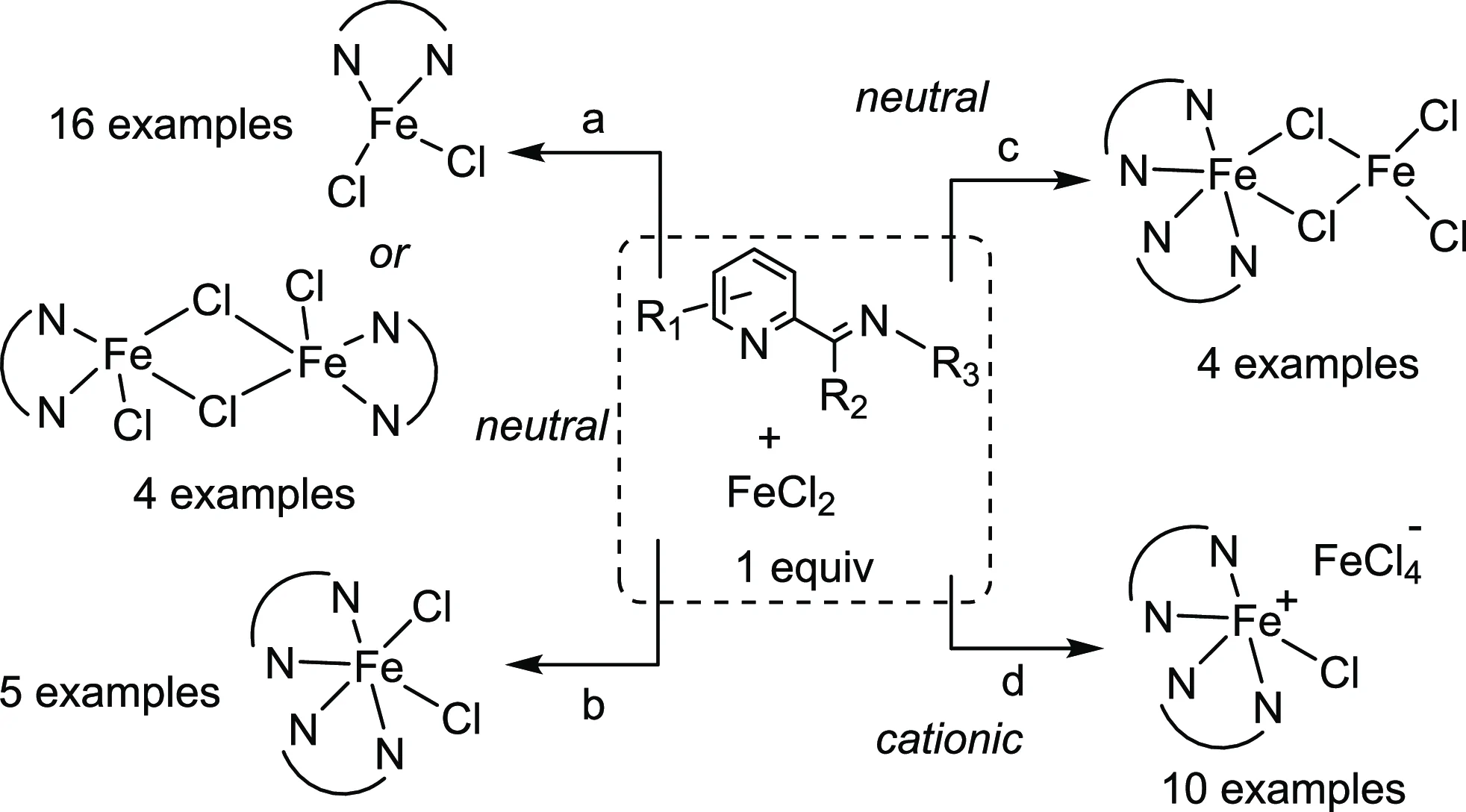
Isolated Molecular Structures of FeCl_2_ Coordinated to ImPys Resolved by Single-Crystal XRD.

In this context, computational chemistry offers a powerful and complementary approach. Density functional theory (DFT) calculations provide direct access to three-dimensional structural landscapes: beyond simple connectivity, modeling allows for the exhaustive exploration of conformational space and coordination geometries, together with spin-state energetics, vibrational fingerprints, and electronic transitions, enabling systematic structure–property correlations to be established. Within this framework, theoretical calculations serve as the bridge between microscopic electronic states and macroscopic observables. Specifically, IR spectroscopy serves as a diagnostic tool for metal–ligand bonding and coordination geometry, while UV–Vis absorption spectra, dominated by metal-to-ligand charge-transfer (MLCT) transitions, provide a window into ligand field energetics and symmetry-breaking distortion. This correlation-based strategy is particularly advantageous when single-crystal XRD is inaccessible, allowing for the characterization of species in the solution or bulk environments. Establishing such a diagnostic framework is crucial as the local coordination environment and electronic configuration are the primary determinants of the catalytic behavior.

The present study aims to investigate a series of ImPy–Fe­(II) complexes through a comprehensive DFT and Time-Dependent Density Functional Theory (TD-DFT) investigation. Emphasis is placed on pseudo-octahedral motifs, which represent the most relevant models for a systematic investigation of spin-state equilibria and ligand-field effects. Our library combines newly developed models with all previously reported mononuclear (ImPy)_2_FeCl_2_ and dinuclear (ImPy)_2_Fe­(*μ*-Cl_2_)­FeCl_2_ complexes. Key structural parameters, including Fe–N bond distances and octahedral distortion metrics (ζ, Σ, θ), are systematically correlated with vibrational properties, MLCT transition energies, UV–Vis spectral features, and frontier orbitals gaps.

Validation of the computational trends against experimental data establishes robust IR and UV–Vis fingerprints for the spectroscopic characterization of pseudo-octahedral ImPy–Fe­(II) complexes. More generally, the methodology developed herein provides a practical framework for spectral assignment and supports the rational design of new ImPy–Fe­(II) catalysts by linking molecular structure, electronic configuration, and spectroscopic response.

## 2 Computational Models and Methodology

### 2.1 Models Selection

The study initiates with a systematic investigation of the bis-ligated complex (*N*,1-diphenyl-1-(pyridin-2-yl)­methanimine)_2_FeCl_2_ (**M1** in Chart [Fig chart1]), first reported by some of us,^12^ and extensively characterized by single-crystal XRD, IR, and UV–Vis spectroscopy. Owing to the extensive experimental data available, **M1** serves as an ideal benchmark for exploring the intrinsic sensitivity of ImPy–Fe­(II) complexes to subtle structural variations. A comprehensive computational analysis was therefore performed on **M1**, encompassing all relevant geometric isomers (Chart [Fig chart2]), and spin-state configurations. The corresponding dinuclear species (**D1**) was also investigated in order to establish a comprehensive reference benchmark. This approach allows to disentangle the effects of isomerism and spin state on key structural parameters, vibrational signatures, and electronic properties, thereby ensuring a coherent interpretation of the complete structural library. Although a complete exploration of the isomeric landscape was performed only for the benchmark complex **M1**, the purpose of this exhaustive analysis was not to establish the preferred coordination geometry of every ligand derivative. Rather, **M1** was used as a reference complex to disentangle the individual effects of isomerism and spin state on the structural descriptors and the corresponding IR and UV–Vis signatures. The broader ligand library was then employed to assess the transferability of these spectroscopic structure–property relationships across chemically related pseudo-octahedral ImPy–Fe­(II) complexes.

**1 chart1:**
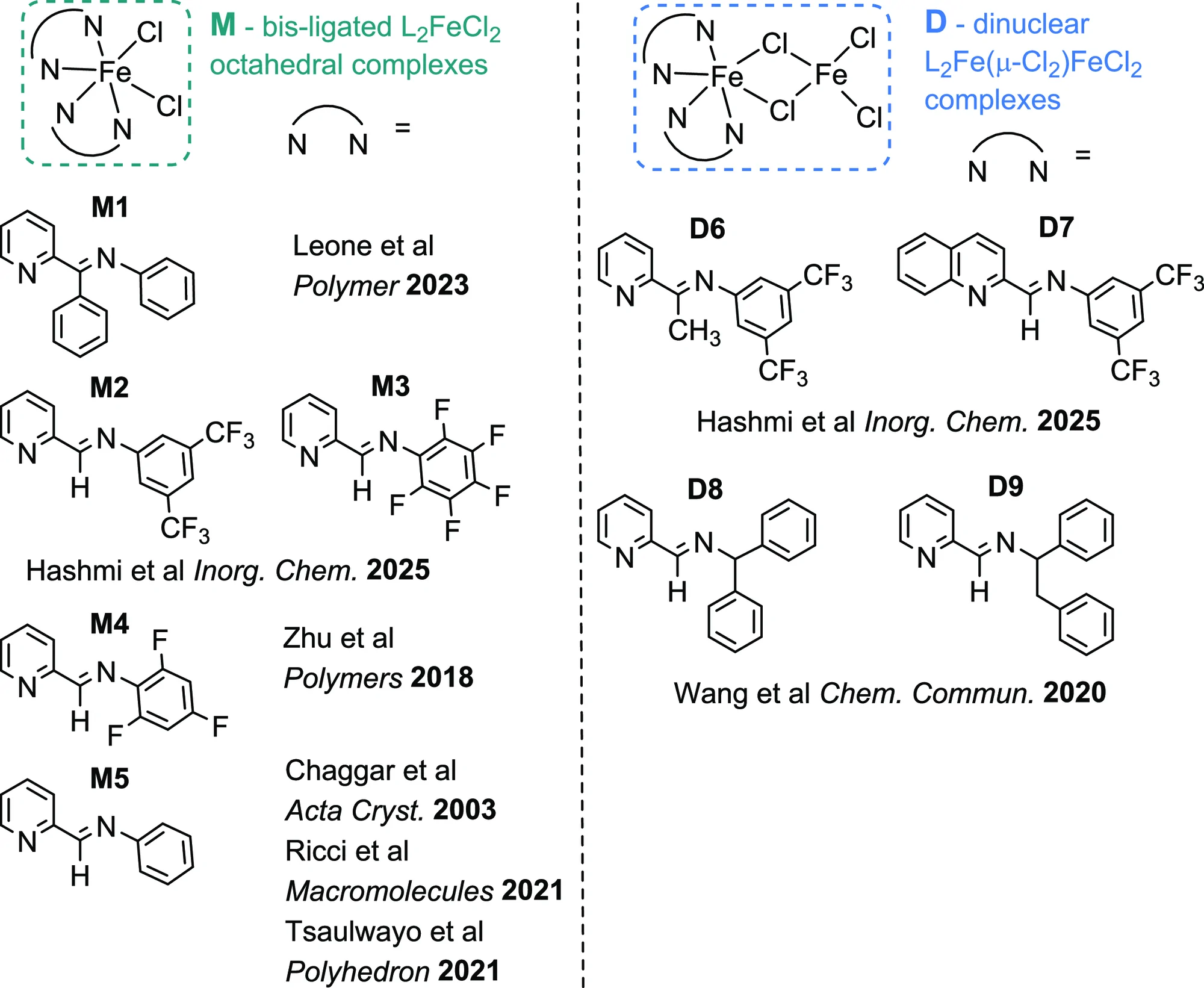
Mononuclear (ImPy)_2_FeCl_2_ (**M**) and Dinuclear (ImPy)_2_Fe­(*μ*-Cl_2_)­FeCl_2_ (**D**) Complexes Investigated in This Study.

**2 chart2:**
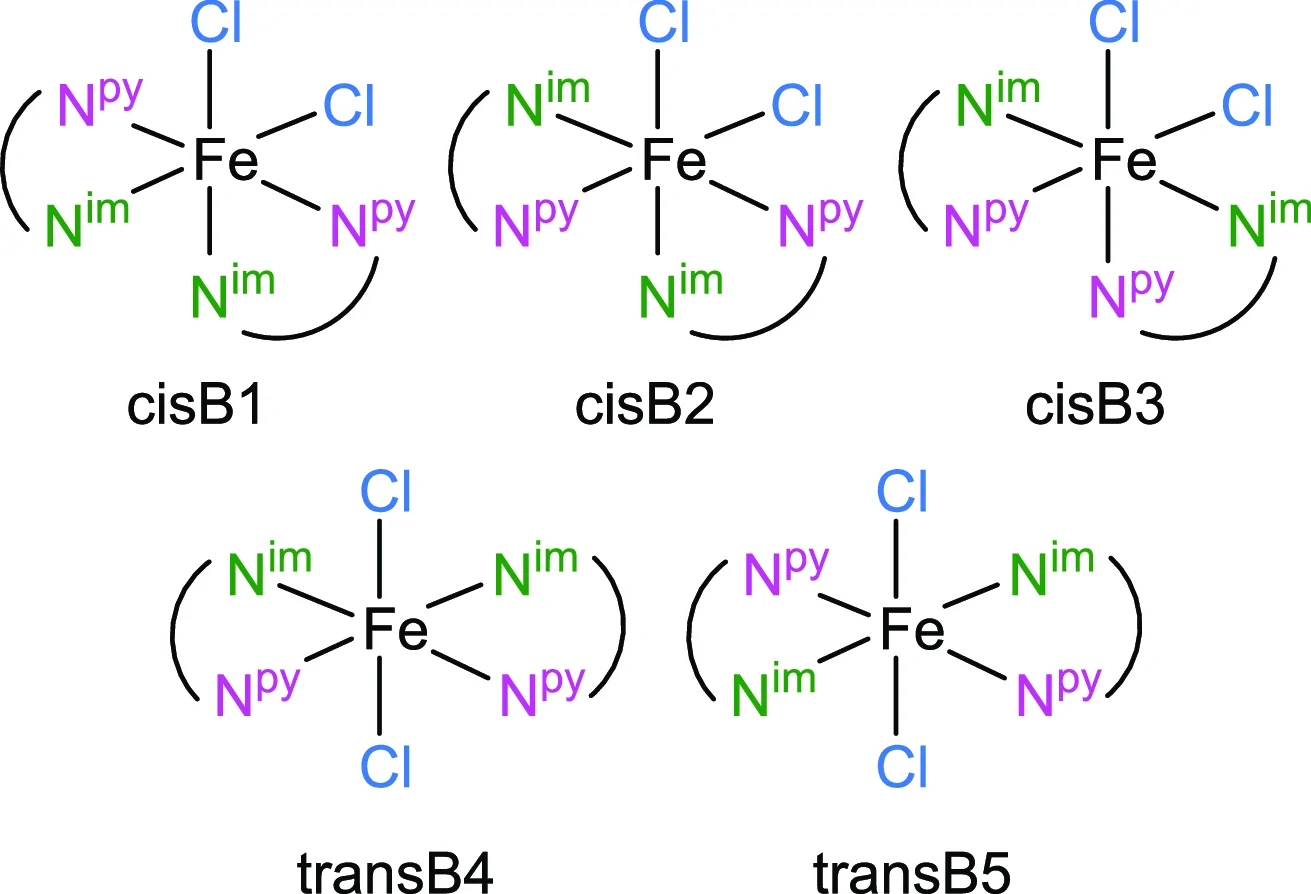
Possible Isomeric Arrangements.

The relative electronic energies of all optimized **M1** and **D1** structures are reported in Table S1. In both the mononuclear and dinuclear series, the experimentally observed ***cisB1*** **-HS** arrangement is predicted to be the thermodynamic minimum owing to a favorable ligand arrangement and a network of intramolecular non-covalent interactions (Figure S1). In contrast, the remaining *cis* and *trans* isomers progressively lose these stabilizing interactions, resulting in higher relative energies.

With this benchmark established, the computational analysis was extended to all previously reported pseudo-octahedral ImPy–Fe dichloride complexes for which structural data are available (Chart [Fig chart1] and Table S2). They comprise both mononuclear (ImPy)_2_FeCl_2_ (designated as **M**) and dinuclear (ImPy)_2_Fe­(*μ*-Cl_2_)­FeCl_2_ (designated as **D**) species, as reported by Hashmi (**M2**, **M3**, **D6**, **D7**),^10^ Wang (**M4**, **D8**, **D9**),^15^ and Chaggar (**M5**) (number indicates specific ligand).^27^ The enclosure of these complexes allowed us to expand the scope of the study and assess whether the trends identified for **M1** are transferable to compounds featuring diverse ligands and structural motifs. In all cases, geometry optimizations were performed using the same computational protocol adopted for **M1**, explicitly considering both LS and HS to ensure methodological consistency across the dataset.

Overall, a total of 37 optimized structures were obtained, corresponding to 37 distinct data points used in the subsequent correlation analyses (Table S3). This statistically meaningful dataset spans a range of ligand substitution patterns, nuclearities, isomeric arrangements, and spin states, while preserving a common pseudo-octahedral coordination motif at the Fe­(II) center.

### 2.2 Computational Details

Calculations were performed using Gaussian 16, Rev. C.01,^28^ at the DFT level employing the unrestricted Kohn–Sham formalism. Geometry optimizations were carried out using initial structures obtained from XRD data, when available. Singlet and quintet complexes were computed for mononuclear complexes, while singlet, quintet, and nonet states were considered for the dinuclear systems. A preliminary benchmark was carried out to identify a computational protocol capable of reproducing the structural and electronic properties of the investigated Fe­(II) complexes. To this end, a representative set of density functionals, including the GGA functional PBE,^29^ and hybrid functionals (B3LYP,^30–33^ B3LYP* featuring a reduced Hartree–Fock exchange contribution of 15%,^34^ and PBE0 ^35^), was employed to evaluate their performance in describing the geometries and electronic structure of the complexes. These approaches were evaluated together with different basis sets (SVP,^36^ def2-SVP,^37^ and def2-TZVP^37^), to assess the effect of basis-set size on the calculated properties. The influence of implicit solvation was also evaluated using both the Conductor-like Polarizable Continuum Model^38,39^ (CPCM) and Solvation Model based on Density^40^ (SMD). The performance of the different computational protocols was evaluated by comparison with the available experimental structural data and relevant spectroscopic observables. Based on this assessment (Table S4), and consistently with previous studies on related Fe­(II) systems,^41^ geometry optimizations were performed using the B3LYP-D3­(BJ) functional, the def2-SVP basis set, and the SMD model with CHCl_3_ as the solvent. Frequency calculations at the same level of theory confirmed that the optimized structures correspond to true minima on the potential energy surface. The simulated IR spectra were obtained by convolution of the calculated transitions with Gaussian functions with a full width at half maximum (FWHM) of 4 cm^–1^ using GaussView.^42^ A scaling factor of 0.96 was also applied to the calculated frequencies.^43,44^

Excited-state calculations were performed using TD-DFT. The accurate reproduction of experimental UV–Vis spectra for transition metal complexes, particularly those of iron, remains a significant challenge for TD-DFT, as extensively documented in the literature.^45,46^ Consequently, several functionals were tested, including TPSSh^47^ (HF exchange = 10%), B3LYP, OPBE,^29,48^ CAM-B3LYP,^49^ M06,^50^ and M06-2X,^50^ in order to evaluate their performance in describing the excited states. The TPSSh/def2-TZVP/SMD­(CHCl_3_) protocol was ultimately adopted as the optimal method, as it demonstrated the best agreement with the experimental spectral profile. The energies of the 50 lowest excited states were computed. From the comparison with the experimental data, the theoretical transitions were convoluted with Gaussian functions using GaussView, applying a FWHM of 2000 cm^–1^ (0.25 eV) and a uniform energy shift of 5000 cm^–1^ (0.62 eV), as suggested in ref 45 and 46. Natural transition orbital^51^ (NTO) analyses were performed for the relevant electronic excitations in order to provide a compact orbital-based description of the excitation character.

### 2.3 Structural Analysis and Distortion Parameters

To quantify deviations from an ideal octahedral geometry, we employed the OCTADIST software,^52^ a specialized tool that provides a standardized framework for evaluating octahedral distortion in organometallic complexes. This choice is crucial because distortion parameters are not uniquely defined across different studies, and ad hoc definitions often lead to inconsistencies when comparing data. By using OCTADIST, we ensure that all metrics are calculated according to a codified and reproducible methodology. For clarity, we distinguish between (i) primary geometrical descriptors, which define the size and specific local features of the iron coordination sphere, and (ii) distortion parameters, which quantify deviations from an ideal octahedron.

#### 2.3.1 Primary Geometrical Descriptors

##### 2.3.1.1 Average Fe-Ligand Distance ⟨*D*⟩

$$\left\langle D\right\rangle \left(Å\right)=\frac{1}{6}\overset{6}{\underset{i=1}{\sum }}d_{i}$$

where *d*_*i*_ represents each individual M–X bond distance. It represents the mean value of the six Fe–ligand bond lengths and reflects the global radial expansion or contraction of the coordination sphere. This parameter is directly linked to the electronic spin state, as LS complexes typically exhibit shorter average bond lengths than HS species.

##### 2.3.1.2 Cl–Fe–Cl Angle

The Cl–Fe–Cl angle is defined as the angle between the two chloride ligands in *cis* or *trans* arrangement. This descriptor does not measure distortion from an ideal octahedron per se, but rather provides a chemically intuitive descriptor of halide positioning that directly affects Fe–Cl orbital overlap and donor orbital stabilization in MLCT transitions.

#### 2.3.2 Distance and Angular Distortion Parameters

##### 2.3.2.1 Distance Distortion ζ:

$$\zeta \left(Å\right)=\overset{6}{\underset{i=1}{\sum }}\left|d_{i}-\left\langle D\right\rangle \right|$$

where *d_i_* is individual M–X bond distance and ⟨*D*⟩ is the average metal-ligand bond distance. This parameter reflects the absolute spread of Fe–ligand distances around ⟨*D*⟩. It captures radial heterogeneity in bond strengths and provides a measure of how uniformly the coordination sphere expands or contracts.

##### 2.3.2.2 Radial Distortion Δ:

$$\Delta =\frac{1}{6}\overset{6}{\underset{i=1}{\sum }}{\left(\frac{d_{i}-\left\langle D\right\rangle }{\left\langle D\right\rangle }\right)}^{2}$$

Δ is defined as the normalized variance of the six Fe–ligand bond lengths and represents a dimensionless measure of radial anisotropy within the coordination sphere. Unlike ⟨*D*⟩, which reflects global size, Δ captures internal asymmetry between shorter and longer bonds, even when the average distance remains unchanged. From a physical standpoint, Δ probes the degree of bond-length anisotropy that can influence d-orbital splitting and donor orbital stabilization.

##### 2.3.2.3 Angular Distortion Σ:

$$\Sigma =\overset{12}{\underset{i=1}{\sum }}\left|90^{{}^{\circ}}-\phi_{i}\right|$$

where *ϕ_i_* represents each *cis* angle around the metal center. Σ captures the deviation of *cis* angles from the ideal 90°, thus probing angular strain within the coordination sphere.

##### 2.3.2.4 Torsional Distortion Θ:

$$\Theta =\overset{24}{\underset{i=1}{\sum }}\left|60^{{}^{\circ}}-\theta_{i}\right|$$

where *θ_i_* is the torsional angle between two opposite triangular faces of the octahedron. Θ quantifies the degree of trigonal twist, indicating whether the geometry tends toward a trigonal prismatic arrangement.

In summary, ⟨*D*⟩ and the Cl–Fe–Cl angle act as primary geometrical descriptors defining global size and local halide positioning, respectively. In contrast, ζ and Δ quantify distance and radial distortions, while Σ and Θ capture angular and torsional deviations from ideal octahedral symmetry. These parameters provide a comprehensive mapping of the coordination sphere, serving as a strategic set of descriptors that can eventually be correlated with vibrational and electronic signatures to decode catalyst behavior.

## 3 Experimental Methods

### 3.1 Characterization

**M1** and **M5** complexes were synthesized according to previously reported procedures.^12,13^ While their molecular structures have been previously described, their vibrational and electronic spectroscopic characterization is reported here for the first time to provide a direct experimental benchmark for comparison with the computational results. All the manipulation and sample handling were carried out under inert atmosphere using a glovebox, unless otherwise stated.

Infrared (IR) spectra were recorded in both the mid-IR (MIR) and far-IR (FIR) regions. MIR spectra of the complexes in the solid state were recorded in the 4000–400 cm^–1^ range using attenuated total reflectance (ATR) mode with a spectral resolution of 2 cm^–1^, on a Bruker Alpha spectrophotometer equipped with a diamond ATR crystal placed directly inside the glovebox. FIR spectra in the 590–100 cm^–1^ range were collected at a spectral resolution of 4 cm^–1^ on powdered samples in ATR mode, using a Bruker Vertex 70 spectrophotometer equipped with a Si beam splitter and a far-IR DTGS detector. For FIR measurements, samples were briefly exposed to air (less than one minute); control experiments confirmed that this short exposure did not result in any detectable degradation of the material.

UV–Vis absorption spectra were recorded in transmission mode using anhydrous CHCl_3_ as solvent (c = 1 × 10^–3^ mol L^–1^). Measurements were carried out directly inside the glovebox using an Avantes portable spectrophotometer equipped with optical fibers. The instrument comprises a dual-lamp radiation source covering the UV and visible spectral regions, a CCD detector, and a set of optical fibers connecting the source to the sample holder and the sample holder to the detector. The spectrophotometer was positioned outside the glovebox and optically coupled to the interior through a dedicated fiber-optic feedthrough. Measurements were performed using quartz (Suprasil) cuvettes with optical path lengths of 1 cm.

## 4 Results and Discussion

### 4.1 Computational Library: Structural, Vibrational, and Electronic Trends

#### 4.1.1 Correlation between Geometrical Parameters

The analysis initially sought to identify systematic trends within the geometric descriptors established in the previous section. Particular attention was devoted to assess whether these parameters provide reliable markers of spin state and whether their trends are affected by nuclearity. The numerical values of all structural descriptors for the optimized structures (including all spin states) and the experimental structures are reported in Tables S3 and S2 of the Supporting Information, respectively.

Initially, the spatial descriptors were evaluated by plotting the distance distortion parameter ζ as a function of the average Fe–ligand bond length, ⟨*D*⟩ (Figure 1a). The data segregate into two distinct clusters for both mononuclear (Mx,) and dinuclear (Dx) complexes. All low-spin (LS) complexes cluster withing a narrow range of short average distances (⟨*D*⟩ ∼ 2.10 Å) and display consistently large distance distortions, with ζ values typically close to ∼1.0–1.1 Å. In contrast, HS complexes are characterized by uniformly elongated Fe–ligand bonds, with ⟨*D*⟩ remaining essentially constant across the entire dataset (⟨*D*⟩ ∼ 2.30 Å). On the other hand, the magnitude of ζ exhibits a clear dependence on nuclearity. Mononuclear HS complexes display relatively small distance distortions (ζ < 0.6 Å), whereas dinuclear HS species show systematically larger ζ values, typically in the 0.7–0.8 Å range. The robustness of these distance-based trends was further validated through a systematic analysis of the reference complex **M1**, for which all relevant *cis* and *trans* isomers were explicitly considered in both LS and HS configurations. However, the clear separation between LS and HS complexes and the nuclearity-dependent behavior of ζ within the HS state remain essentially unaffected.

**Figure 1:**
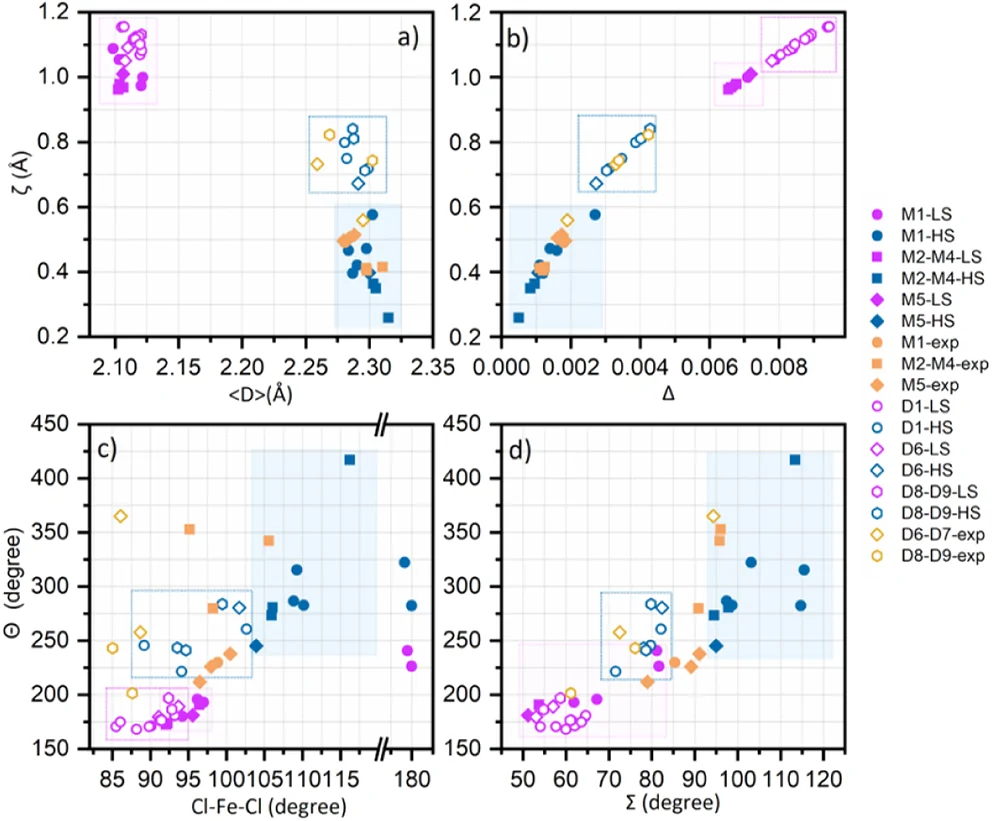
Correlation between geometrical descriptors for the computational library of pseudo-octahedral ImPy–Fe complexes investigated in this work. Panels (a) and (b) report radial descriptors: (a) distance distortion parameter ζ as a function of the average Fe–ligand bond length ⟨*D*⟩; (b) correlation between ζ and the normalized radial distortion parameter Δ. Panels (c) and (d) report angular descriptors: (c) torsional distortion parameter Θ as a function of the Cl–Fe–Cl angle; (d) correlation between the angular distortion parameters Θ and Σ. Color code: pink = low-spin (LS); blue = high-spin (HS); orange = experimental data. The **M** complexes were simulated in both LS and HS states. For the **D** complexes, containing two iron centers, three spin-state combinations were considered: LSLS, LSHS, and HSHS. Pink and blue markers denote complexes in which the iron center coordinated to the ligands is in the LS and HS iron centers, respectively, regardless of the spin state of the second metal center (Table S2 and S3).

To provide a more comprehensive description of the radial deviations, we examined the normalized radial distortion parameter Δ as a function of ⟨*D*⟩ (Figure S2). Remarkably, Δ vs ⟨*D*⟩ exhibits the same qualitative behavior observed for ζ vs ⟨*D*⟩: LS complexes cluster at short average bond lengths and large Δ values, whereas HS species occupy the elongated bond-length region with systematically smaller Δ values. This congruent trend confirms that both ζ and Δ consistently capture the radial expansion associated with the spin-state transition.

Although ζ and Δ display nearly identical separation of LS and HS complexes when plotted against ⟨*D*⟩, they differ in their mathematical definition: ζ is based on the absolute deviation from the mean bond length, whereas Δ corresponds to a normalized quadratic variance. Consequently, when ζ is plotted directly as a function of Δ (Figure 1b), a strong but non-linear correlation emerges. The resulting trend displays a monotonic curved behavior, which is fully consistent with the mathematical relationship between the two descriptors. Importantly, the monotonic correlation confirms that ζ and Δ provide internally consistent measures of radial heterogeneity within the coordination sphere, with ζ reflecting the absolute spread of Fe–ligand distances around ⟨*D*⟩, and Δ placing greater emphasis on larger deviations through quadratic weighting.

Overall, these results demonstrate that distance-based descriptors provide a robust criterion for spin-state identification. Furthermore, they exhibit sensitivity to secondary structural features such as nuclearity, while remaining largely invariant to isomeric rearrangements. The average Fe–ligand bond length ⟨*D*⟩ acts as a primary marker of electronic configuration, while ζ and Δ quantify the extent and nature of bond-length distribution.

We next analyzed angular distortions following an analogous approach: we first considered the Cl–Fe–Cl angle as a chemically intuitive local descriptor of halide positioning, and subsequently examined how this parameter relates to the global torsional and angular distortion metrics. The Cl–Fe–Cl angle was selected because it displays the largest variability among *cis* angles and shows the clearest correlation with octahedral distortion, whereas analogous analyses involving N–Fe–N or N–Fe–Cl angles do not reveal meaningful trends. Figure 1c shows the relationship between the torsional distortion parameter Θ and the Cl–Fe–Cl angle. When the full dataset is considered, *cis* and *trans* isomers behave differently: *trans* complexes display Cl–Fe–Cl angles close to 180°, while spanning a broad range of Θ. In contrast, restricting the analysis to *cis* complexes, a clear and nearly monotonic correlation emerges: opening of the Cl–Fe–Cl angle is accompanied by a progressive increase of Θ, indicating that torsional distortion is strongly driven by halide–halide repulsion and the degree of chloride opening around the metal center. Within this subset, distinct trends are observed for mononuclear (**Mx**) and dinuclear (**Dx**) complexes. Dinuclear complexes systematically populate the smaller Cl–Fe–Cl region, reflecting the geometric constraints imposed by chloride bridging, whereas mononuclear species extend to larger Cl–Fe–Cl angles, consistent with their greater structural flexibility. Notably, although LS and HS complexes occupy different angular domains in this representation, the separation is less abrupt than for the radial descriptors. The data form recognizable clusters, but with broader dispersion and partial overlap, particularly within the HS manifold.

Having established the connection between local halide positioning and torsional distortion, we then examined the relationship between the two global angular distortion parameters, Θ vs Σ (Figure 1d). The same trend toward broader dispersion is observed in the θ–Σ plot. While LS complexes remain relatively well clustered in the low-distortion region (Σ = 50–70°, Θ = 150–200°), HS species span a significantly wider angular space, leading to partial overlap between the two spin manifolds. A small subset of LS *trans* isomers is slightly shifted toward larger Σ and Θ values, yet remains clearly separated from the mononuclear HS isomers. This tight clustering indicates that the low-spin electronic configuration imposes a strongly constrained octahedral geometry, which is only weakly perturbed by ligand topology or by the presence of a second metal centre. In contrast, HS complexes populate a broader region of the Θ–Σ space, with a clear differentiation emerging between mononuclear and dinuclear species. Dinuclear HS complexes are characterized by comparatively smaller distortion, clustering at intermediate Σ and Θ values, whereas mononuclear HS complexes extend toward significantly larger distortion (Σ = 100–120°, Θ up to 420°). This behavior highlights that, within the HS manifold, angular and torsional distortions are strongly modulated by nuclearity.

Taken together, these results demonstrate that angular and torsional distortions do not simply mirror spin-state differences, but capture subtler geometric variations that arise from interplay between electronic configuration, ligand architecture, nuclearity and isomerism. While distance-based descriptors, such as ⟨*D*⟩, ζ and Δ, provide a robust and largely unambiguous criterion for spin-state identification, angular metrics reveal how the coordination sphere adapts to steric and topological constraints, particularly within the more flexible high-spin regime. In this context, Σ and Θ act as global descriptors of octahedral deformation, capturing nuclearity-dependent trends that are not accessible through bond-length analysis alone.

From the perspective of spin-state assignment, all LS complexes occupy a narrowly defined angular region characterized by relatively small Cl–Fe–Cl angles (∼85–100°) and low torsional distortions (Θ < 200°, Σ < 70°) independently of nuclearity, with only minor deviations observed for *trans* isomers. In contrast, HS species span a much broader region of the angular space, extending to larger Cl–Fe–Cl angles and higher Θ and Σ values, and partially overlapping with the LS domain in the intermediate angle regime (∼90–100°). This pronounced asymmetry endows the Cl–Fe–Cl angle with a practical diagnostic value: while intermediate angles cannot unambiguously discriminate between LS and HS states, strongly compressed Cl–Fe–Cl angles are reliable indicators of LS configurations. In this sense, the Cl–Fe–Cl angle acts as a necessary, but not sufficient, structural marker for the LS state; its predictive power is significantly enhanced when integrated with distance-based descriptors such as ζ.

To further evaluate the robustness of the identified descriptor–property relationships, an unsupervised principal component analysis (PCA) was performed using the complete set of structural descriptors, including the radial distortion parameter (⟨*D*⟩), the ζ parameter, the angular distortion parameter (Σ), the torsional distortion parameter (Θ), and the Cl–Fe–Cl angle. The PCA analysis independently confirms that the structural descriptors highlighted by the chemically driven interpretation represent the main sources of variability within the dataset. In particular, the first principal componen (PC1) is mainly determined by the different distortion parameters, with comparable contributions from ⟨*D*⟩; (21.3%), ζ (23.3%), Σ (24.4%), and Θ (23.6%), whereas the Cl–Fe–Cl angle provides a minor contribution (7.4%). Conversely, the second principal component (PC2) is dominated by the Cl–Fe–Cl angle (82.8%), indicating that this descriptor captures an independent structural component associated with the relative orientation of the coordination environment. These first two principal components account for 94.50% of the total variance within the dataset, with PC1 explaining 77.46% and PC2 17.05% of the variance (Table S5, S6 and Figure S3). The PCA distribution separates the three structural families identified through the clustering analysis, consisting of 19 LS-*cis*, 14 HS-*cis*, and 4 *trans* geometries. The close agreement between the statistical analysis and the chemically interpretable descriptor-based approach provides independent support for the robustness of the proposed structure–property relationships.

The **D6–D9** complexes provide a useful test of the sensitivity of the structural descriptors to asymmetric spin-state distributions in dinuclear systems. For these complexes, the OCTADIST analysis necessarily refers to the six-coordinate Fe center bound to the ImPy ligands. The distance-based descriptors consistently place the experimentally characterized **D6–D9** structures within the HS region, in agreement with the assignment of the ligand-coordinated Fe center as high spin. However, **D6–D7** and **D8–D9** cannot be clearly separated on this basis, because the spin state of the second Fe center, the four-coordinate one, has only a limited influence on the octahedral descriptors calculated for the ligand-bound Fe site. The angular descriptors show a broader dispersion and likewise do not provide a unique criterion for distinguishing HS–HS from HS–LS dinuclear species. Thus, the structural descriptors reliably identify the spin state of the pseudo-octahedral Fe center, but they do not directly report the electronic configuration of the second pseudo-tetrahedral Fe center.

#### 4.1.2 Vibrational Signatures

To assess whether the structural trends identified in the previous section translate into experimentally accessible fingerprints, we analyzed the simulated infrared (IR) spectra of representative complexes from the computational library. Particular emphasis was placed on evaluating the sensitivity of IR spectroscopy to isomerism, spin state, and ligand environment, together with identifying diagnostic vibrational regions that can be directly related to well-defined structural descriptors. We first focused on the reference systems **M1** and **D1**, for which all relevant geometric isomers were explicitly considered in both LS and HS configurations. This exhaustive dataset provides a stringent benchmark for probing how subtle variations in coordination geometry and electronic configuration are reflected in the vibrational response.

The simulated spectra (Figure 2) exhibit pronounced differences across both isomers and spin states, indicating that IR spectroscopy is highly responsive to changes in local bonding environments, even when the overall coordination motif remains pseudo-octahedral. Across all **M1** (Figure 2a) and **D1** (Figure 2b) isomers, three spectral regions emerge as particularly informative.

**Figure 2:**
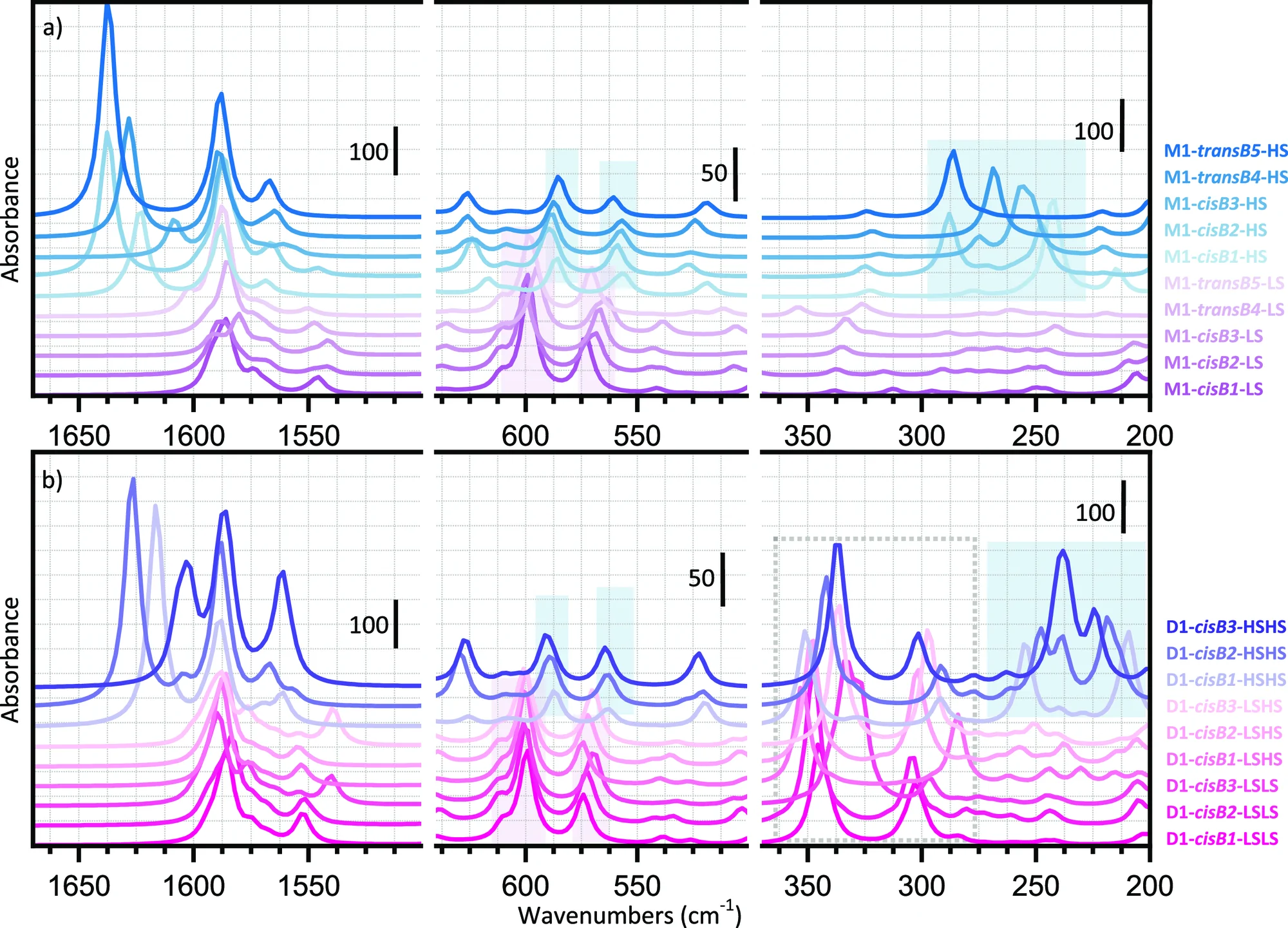
Simulated IR spectra for complexes **M1** (a) and **D1** (b), including all relevant geometric isomers and both low-spin (LS) and high-spin (HS) electronic configurations. Three diagnostic spectral regions are highlighted: i) 1665–1500 cm^–1^, dominated by ligand-centered vibrations; ii) 635–500 cm^–1^, dominated by N–Fe–N torsional modes; iii) 365–200 cm^–1^, corresponding to Fe–Cl stretching modes.

The high wavenumber region (1650–1550 cm^–1^) is dominated by ligand-centered vibrations, particularly the C=N stretching modes of the imino bridges, which are strongly affected by coordination to FeCl_2_. While these bands are present in all complexes, their position and relative intensity vary significantly among different geometries. In particular, selected HS isomers (the two *trans* configurations and *cisB2*) display a marked shift of the C=N stretching mode toward higher wavenumbers, consistent with a weakening of metal-ligand back-donation and a more localized imine character. In contrast, LS complexes show a narrower distribution of band positions in this region, reflecting a more uniform and rigid Fe–N bonding environment.

A second diagnostic region is located between approximately 635 and 500 cm^–1^, where torsional modes involving the N–Fe–N fragment dominate the spectra. These vibrations are highly sensitive to Fe–N bond lengths and, consequently, they provide a robust marker of the spin state. LS complexes, characterized by shorter Fe–N distances and reduced structural flexibility, consistently exhibit these modes at higher wavenumbers. Conversely, HS species display a systematic downshift, reflecting elongated Fe–N bonds and increased vibrational softness. Importantly, this trend is preserved across all isomers of **M1** and **D1**, underscoring the reliability of this spectral region for spin-state discrimination even in the presence of significant isomeric variability.

Finally, the low wavenumber region (400–200 cm^–1^) is dominated by Fe–Cl stretching modes, which are particularly prominent for HS complexes. In this range, both symmetric and antisymmetric ν­(Fe–Cl) vibrations are observed, with their wavenumbers reflecting the coordination environment and relative positioning of the chloride ligands. More open or tetrahedral-like FeCl_2_ arrangements (such as those found in the **D1** complexes) contribute at higher wavenumbers (370–270 cm^–1^), whereas more constrained octahedral environments shift these modes to lower wavenumbers (below 270 cm^–1^, dotted regions in Figure 2). LS complexes, by contrast, display only weak bands in this region, consistent with the reduced amplitude of Fe–Cl stretching motions in a more rigid coordination sphere.

To evaluate the generality of these observations, the analysis was extended to complexes featuring different ImPys (**M2**–**M5**, **D6**, **D8** and **D9**; Figure S4). Although the overall spectral profiles are strongly influenced by ligand substitution, the same qualitative trends identified for **M1** and **D1** are retained. The calculated IR spectra of **D6–D9** further support the general trends identified for reference **D1**. In all cases, the vibrational response is primarily governed by the spin state of the pseudo-octahedral Fe center coordinated to the ImPy ligands. Consequently, **D6–D9** exhibit the same characteristic evolution of the N–Fe–N torsional modes and the ν­(Fe–Cl) region observed for the corresponding HS and LS configurations discussed above. No additional vibrational fingerprint specifically associated with the spin state of the second pseudo-tetrahedral Fe center could be identified.

Overall, these results demonstrate that IR spectroscopy provides a direct and sensitive probe of local structural variations in ImPy–Fe­(II) complexes. The simulated spectra reveal clear and transferable qualitative markers for both spin state and coordination environment, enabling discrimination between LS and HS configurations as well as among different geometric arrangements. At the same time, absolute band positions should not be overinterpreted, as they are inherently affected by phase effects, solvation, and anharmonicity limitations of the DFT approach. Within these well-known limitation, however, the relative trends and systematic shifts observed across the computational library remain robust, supporting the use of IR spectroscopy as a reliable diagnostic tool for structure-informed interpretation of ImPy–Fe­(II) complexes in the absence of crystallographic data. These diagnostic vibrational signatures form the basis of the spectroscopic workflow presented in Section 4.1.4.

To complement this analysis, a multiple linear regression (MLR) analysis was performed using the calculated ν­(C=N) stretching frequency as response variable. A broad pool of structural, electronic, and steric descriptors was considered, including the octahedral distortion parameters (⟨D⟩, ζ, Δ, Σ, and Θ), the Cl–Fe–Cl angle, the donor–acceptor energy gap (ΔE), the Mulliken^53^ and the Atomic Polar Tensor (APT)^54^ charges on the Fe center, the average Fe–N_py_, Fe–N_im_, and Fe–Cl bond distances, and the buried volume (%V_Bur_)^55^ as an explicit steric descriptor. Details of the regression models are provided in the Supporting Information (Table S7 and Figure 3).

**Figure 3:**
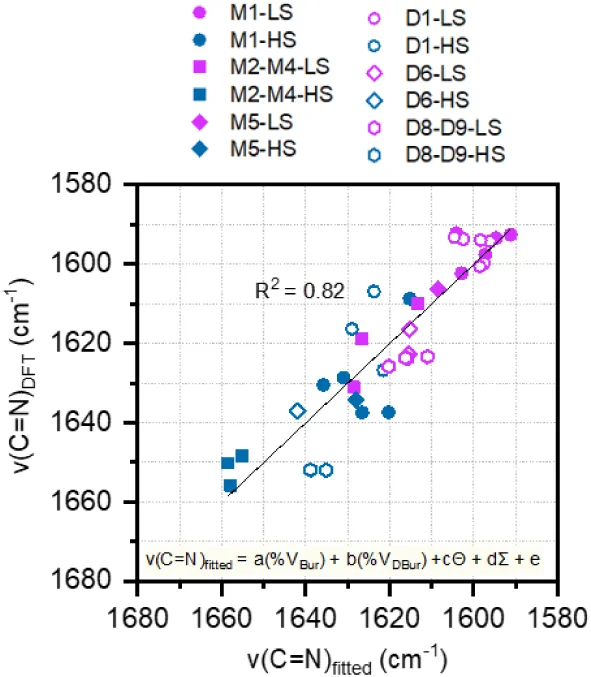
Multiple linear regression analysis of the ν­(C=N) stretching wavenumber. Correlation between the DFT-calculated and MLR-predicted ν­(C=N) values. The regression model combines the buried volume (%V_Bur_ and %V_DBur_), the torsional distortion parameter (Θ), and the angular distortion parameter (Σ). The solid line represents the linear regression.

The optimal regression model included the buried volume descriptors %V_Bur_ and %V_DBur_,^56^ which represent the fraction of the coordination sphere occupied by the ligand within the first coordination sphere and within a spherical crown surrounding the first coordination sphere of the metal center, respectively, the torsional distortion parameter (Θ), and the angular distortion parameter (Σ), yielding an R^2^ of 0.82. Notably, the explicit steric descriptor alone already accounts for most of the explained variance (R^2^ = 0.73), indicating that steric effects are the primary factor governing the ν­(C=N) stretching frequency, while the geometric distortion parameters provide a secondary refinement of the model. The positive correlation between %V_Bur_ and the ν­(C=N) stretching frequency can be rationalized by considering that larger steric demand may weaken the Fe–N interactions, leading to increased Fe–N bond distances and reduced metal-to-ligand electronic communication. As a consequence, the C=N bond retains a larger double-bond character, resulting in an increase of its force constant and a shift of the ν­(C=N) stretching band toward higher wavenumbers.

#### 4.1.3 UV–Vis Signatures

Having established robust correlations between structural descriptors and vibrational signatures, we next investigated whether electronic transitions provide complementary insight into the interplay between coordination geometry and electronic structure. Across the computational library, the simulated spectra display two distinct regions (Figure 4). Intense absorption bands above 20000 cm^–1^ are attributed to ligand-centered π-π* transitions and are largely insensitive to spin state or coordination geometry. In contrast, the lower-energy region (20000–10000 cm^–1^), dominated by MLCT transitions, shows pronounced variations across the dataset. Within the TD-DFT framework, LS complexes are associated with more intense (molar extinction coefficients ε ∼ 10^4^ M^–1^cm^–1^) and higher-energy MLCT bands, whereas HS species exhibit weaker (ε < 0.5 10^4^ M^–1^cm^–1^) and systematically red-shifted transitions. The same qualitative behavior is observed for the asymmetric dinuclear complexes **D6–D9 (** Figure 4d). Despite the different electronic configurations considered (LSLS, LSHS and HSHS), the calculated MLCT transitions are primarily governed by the pseudo-octahedral Fe center coordinated to the ImPy ligands. Accordingly, the spectra follow the same general trends identified for the mononuclear complexes, with LS configurations displaying more intense, blue-shifted transitions and HS configurations weaker, red-shifted absorptions. No additional spectroscopic fingerprint specifically associated with the spin state of the second pseudo-tetrahedral Fe center could be identified within the present dataset.

**Figure 4:**
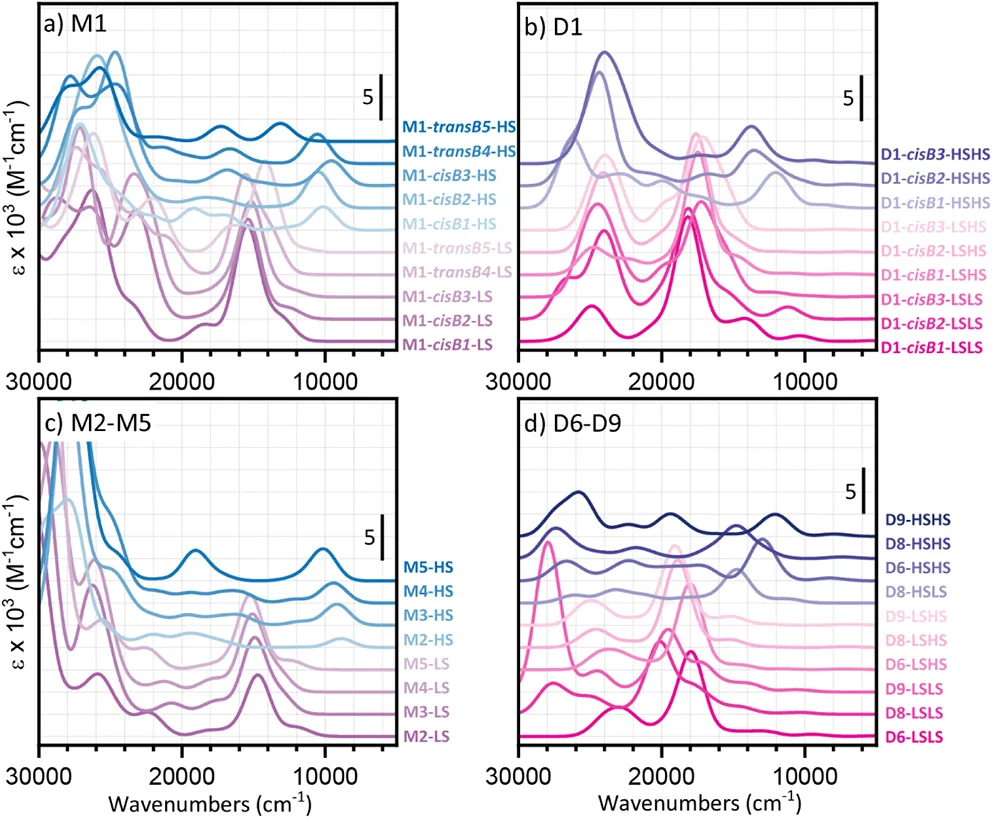
Simulated UV–Vis spectra for the computational library of pseudo-octahedral ImPy–Fe complexes investigated in this work, separated according to nuclearity. Panels (a) and (c) report mononuclear complexes (**Mx**), while panels (b) and (d) show dinuclear complexes (**Dx**) High-energy absorptions (>20000 cm^–1^) correspond to ligand-centered π–π* transitions, while lower-energy bands (20000–10000 cm^–1^) are assigned to MLCT transitions.

To rationalize the origin of these trends, we analyzed the natural transition orbitals (NTOs) involved in the MLCT transitions. As illustrated in Figure 5a for the representative complex **M1**, the donor orbital is consistently localized on the FeCl_2_ fragment, with predominant metal d-character and a non-negligible contribution from the chloride ligands. In contrast, the acceptor orbital is ligand-centered, corresponding to the π* system of the Impy framework. This donor-acceptor pattern is conserved across all complexes and spin states investigated, indicating that the MLCT transitions share a common electronic origin throughout the dataset. A systematic analysis of the frontier orbital energies across the entire dataset (Figure S5 and Figure S6) reveals a clear and robust trend: the acceptor energy is essentially constant (Figure 5c), reflecting its ligand-centered nature, whereas the donor energy varies significantly as a function of both spin state and structural distortion (Figure 5a). In particular, LS complexes consistently exhibit a more stabilized donor orbital relative to HS species, resulting in a larger donor–acceptor gap. Conversely, HS complexes display a destabilized donor orbital and a reduced gap, qualitatively consistent with the red-shifted MLCT transitions observed in the simulated spectra.

**Figure 5:**
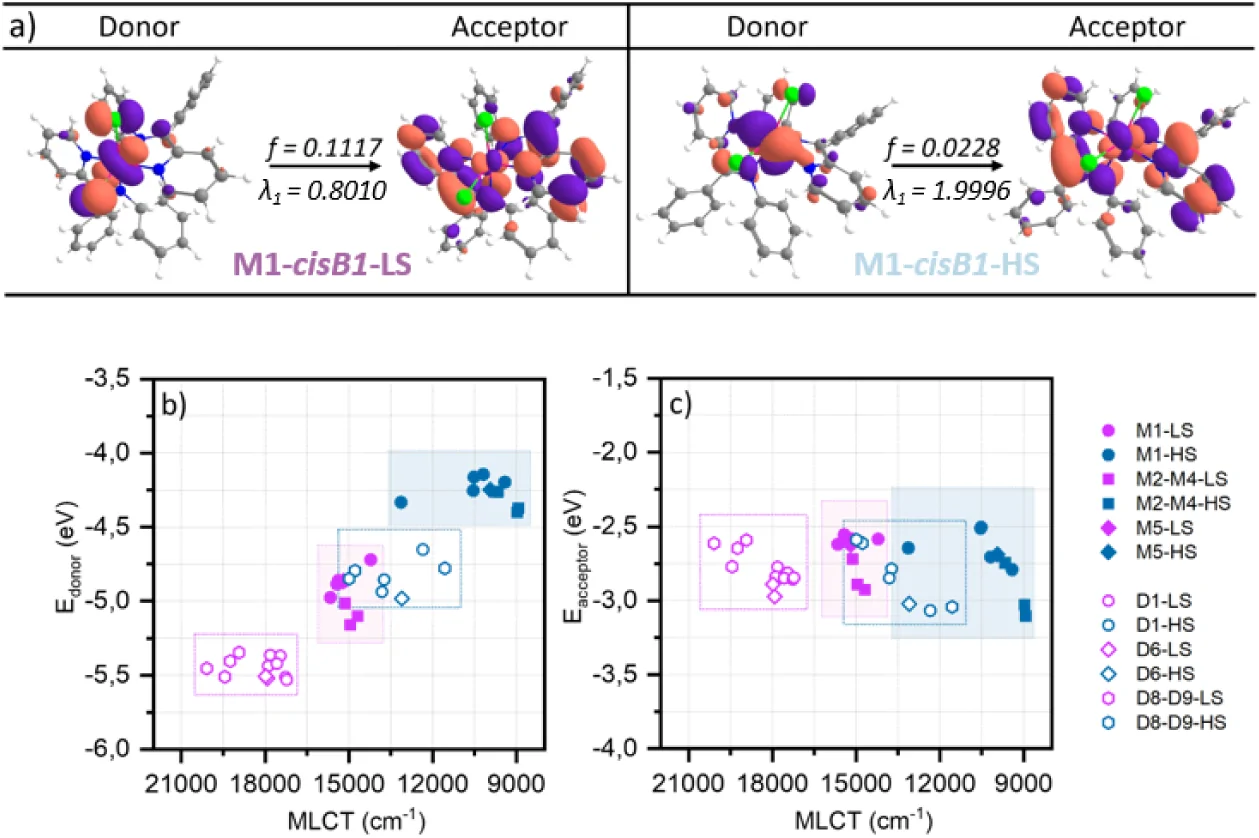
(a) Computed Natural Transition Orbitals (NTOs) for **M1-** *cisB1* **-LS** (excited state 5) and **M1-** *cisB1* **-HS** (excited state 4), with corresponding oscillator strengths (*f*) and dominant NTO pair weights (*λ_1_*). NTO analyses were also performed for species **D1-** *cisB1* and are reported in the Supporting Information (Figure S5). (b) Correlation between the donor orbital energy and the MLCT transition energy. (c) Correlation between the acceptor orbital energy and the MLCT transition energy.

Importantly, this trend is not dictated by spin state alone, but rather reflects the underlying spin-dependent structural features. To further elucidate this point, we examined the relationship between MLCT energies and the structural descriptors identified in Section 4.1.1 (Figure 6). A clear monotonic trend is observed between the radial distortion parameter Δ and the MLCT energy: increasing Δ stabilizes the donor orbital and leads to a blue shift of the MLCT transition (Figure 6a). In contrast, the torsional distortion parameter Θ shows an opposite effect: increasing Θ destabilizes the donor orbital and induces a red shift of the MLCT band. While the Δ−MLCT correlation is nearly linear across the dataset, the Θ–MLCT relationship is broader and less strictly monotonic. This indicates that while torsional distortions play a key role, the global electronic structure is modulated by a more complex interplay of multiple geometric factors. These correlations demonstrate that structural distortions are closely associated with the donor orbital energy, reflecting the metal–ligand overlap and ligand-field splitting. Within this framework, variations in MLCT energy are associated with changes in the energetic position of the donor level, while the acceptor orbital remains largely unaffected (Figure 5b, c and Figure S6).

**Figure 6:**
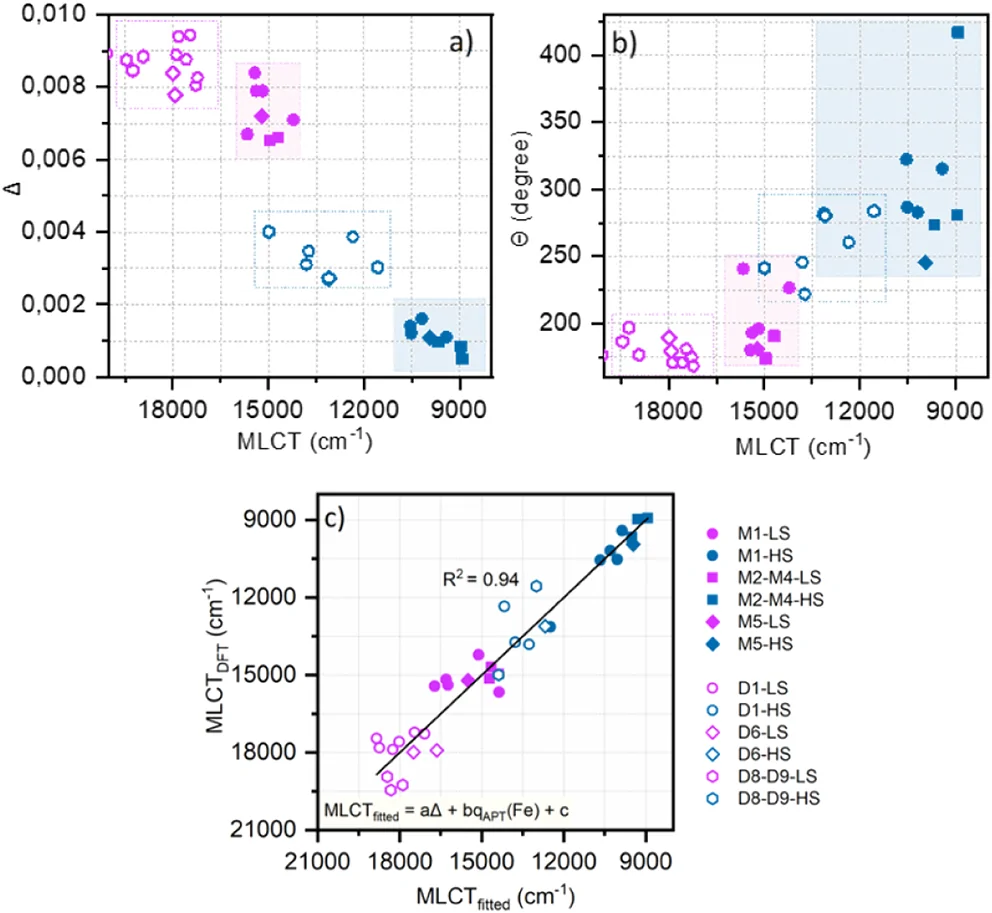
Correlation between (a) MLCT energy and Fe–ligand distance; (b) MLCT energy and angular distortion; (c) correlation between DFT-calculated and MLR-predicted MLCT transition energies using the optimal regression model based on Δ and the Fe APT charge. The solid line represents the linear regression.

The same MLR regression strategy reported above was applied to the calculated MLCT transition energy as response variable (Table S8 and Figure 6c). The best model combines the Δ distortion parameter with the Fe APT charges, increasing the coefficient of determination to R^2^ = 0.94 relative to the univariate correlations discussed above (R^2^ = 0.88), whereas the explicit steric descriptor (%V_Bur_) makes only a negligible contribution. The improvement obtained upon inclusion of the Fe APT charge indicates that, in addition to the geometric distortion of the coordination sphere, the electronic polarization of the metal center plays a significant role in determining the MLCT transition energy. The negligible contribution of the explicit steric descriptor (%V_Bur_) further suggests that steric effects influence the spectra primarily through the geometric distortion they induce rather than through an independent contribution.

#### 4.1.4 Spectroscopic Interpretation Workflow

The computational library developed in this work allows a workflow for the spectroscopic interpretation of pseudo-octahedral ImPy–Fe­(II) complexes to be proposed. Distance-sensitive observables, such as the N–Fe–N torsional modes and the MLCT transition, primarily probe the electronic spin-state, whereas the ν­(Fe–Cl) stretching region provides complementary information on the local chloride coordination environment and the possible presence of tetrahedral-like Fe centers. No individual spectroscopic feature uniquely identifies the molecular structure; however, the combined interpretation of IR and UV–Vis fingerprints considerably narrows the range of plausible coordination environments and spin-state distributions. The principal spectroscopic signatures emerging from the present study are summarized in Table 1.

**Table 1:** Diagnostic Spectroscopic Signatures Forming the Basis of the Workflow Proposed for the Interpretation of Pseudo-Octahedral ImPy–Fe­(II) Complexes^a^

Spectroscopic feature	Diagnostic signature	Structural information	Limitation
ν(Fe–Cl) (270–370 cm^–1^)	Intense bands	Tetrahedral-like FeCl_2_ unit; strong indication of dinuclear complexes	Monoligated FeCl_2_ species may also contribute
ν(Fe–Cl) (<270 cm^–1^)	Intense bands	Terminal Cl bound to pseudo-octahedral HS Fe(II)	Only applicable to HS species
N–Fe–N torsional modes (∼635–500 cm^–1^ region)	LS bands systematically blue-shifted within the same ligand family	Spin-state marker	Ligand-dependent wavenumbers
MLCT (10000–20000 cm^–1^)	LS: blue-shifted, intense; HS: red-shifted, weaker	Spin-state marker	Relative trends more reliable than absolute energies
Combined IR and UV–Vis	Consistent Fe–N, Fe–Cl and MLCT signatures	Structural assignment of the ligand-coordinated Fe center (including nuclearity when supported)	Diagnostic, not standalone

a The table summarizes the characteristic IR and UV–Vis fingerprints, the structural information they provide, and the principal limitations of each diagnostic criterion.

The diagnostic signatures summarized in Table 1 can be integrated into a practical workflow for the interpretation of pseudo-octahedral ImPy–Fe­(II) complexes. The strategy is based on the progressive extraction of structural information from complementary spectroscopic observables, following the sequence outlined below. Whenever the spectroscopic evidence does not allow a unique structural assignment, comparison with the calculated spectra of the most plausible structural models can provide additional support for the final interpretation.

##### 4.1.4.1 Step 1. Identification of Tetrahedral-like FeCl_2_ Units

The interpretation should start from the ν­(Fe–Cl) region, which provides the most direct information on the coordination geometry of the FeCl_2_ unit. The presence of intense ν­(Fe–Cl) bands displaying a characteristic pattern between 270 and 370 cm^–1^ demonstrates that at least one pseudo-tetrahedral FeCl_2_ coordination unit is present in the sample. This spectroscopic fingerprint is associated with the local geometry of the FeCl_2_ fragment rather than with a specific molecular structure. Within the chemical space considered here, this observation implies such a fingerprint is characteristic of chloride-bridged dinuclear complexes containing one pseudo-octahedral and one pseudo-tetrahedral Fe center. However, the same vibrational signature is also expected for any species containing a pseudo-tetrahedral FeCl_2_ unit, including monoligated FeCl_2_ complexes, which are outside the scope of the present computational study. Therefore, this spectral region should be regarded as a probe of the local FeCl_2_ geometry rather than as an unambiguous indicator of nuclearity.

Conversely, the observation of intense ν­(Fe–Cl) bands below 270 cm^–1^ provides strong evidence for the presence of high-spin Fe­(II) centers in pseudo-octahedral coordination, since the corresponding LS species exhibit only weak contributions in this region. Likewise, the absence of intense bands below 270 cm^–1^ rules out the presence of pseudo-octahedral HS Fe­(II) centers as major components of the sample.

##### 4.1.4.2 Step 2. Identification of Multiple Spectroscopic Species

The N–Fe–N torsional region (approximately 635–500 cm^–1^) can then be inspected to evaluate whether the spectrum arises from a single spectroscopic species or from the coexistence of multiple species exhibiting different Fe–N bond lengths and spin states. Rather than focusing on the number of bands, the analysis should consider the overall vibrational pattern characteristic of the ligand. When a second set of bands reproducing the same spectral pattern is observed, but systematically shifted by only a few cm^–1^, this provides strong evidence for the coexistence of different molecular species. Once multiple species have been identified, the direction of the frequency shift can be used to infer their spin state. Within a given ligand family, the pattern occurring at higher wavenumbers corresponds to shorter Fe–N bonds and is therefore associated with LS centers, whereas the lower-wavenumber counterpart is characteristic of HS configurations. Because the absolute wavenumbers and the detailed vibrational pattern depend on the ligand framework, this analysis should always be carried out by comparison with an experimentally characterized reference compound bearing the same ligand scaffold or with calculated spectra of the plausible structural models. Note that the N–Fe–N torsional region represents the most general structural fingerprint identified in this work. Additional ligand-centered vibrations may provide further discrimination for specific ligand families.

##### 4.1.4.3 Step 3. Spin-State Confirmation by UV–Vis Spectroscopy

The final step consists in analyzing the MLCT region of the UV–Vis spectrum (10000–20000 cm^–1^), which provides complementary information on the electronic configuration. Both LS and HS complexes exhibit MLCT transitions within this energy range; therefore, the presence of a band alone cannot be used to discriminate between the two spin states. Instead, the transition energy and intensity should be considered together. Within the investigated computational library, LS complexes display systematically more intense (ε ≈ 10^4^ M^–1^cm^–1^) and blue-shifted MLCT transitions, whereas HS species exhibit weaker (ε < 0.5 × 10^4^ M^–1^cm^–1^) at lower energies. Although these trends are robust, the overlap between the two spin manifolds prevents the definition of universal energy thresholds. Moreover, quantitative interpretation of the molar extinction coefficient requires complete solubility, accurate concentration measurements, and the absence of significant speciation in solution. Consequently, the UV–Vis spectrum provides the final consistency check of the spectroscopic workflow. Its combined interpretation with the IR fingerprints enables the selection of the most plausible coordination environment and spin-state distribution within the investigated family of pseudo-octahedral ImPy–Fe­(II) complexes.

### 4.2 Experimental Data in the Context of the Computational Landscape

To assess how experimentally characterized ImPy–Fe­(II) complexes relate to the computational framework developed in the previous sections, we first projected all available crystallographic data onto the structural correlation plots shown in Figure 1. In total, the nine pseudo-octahedral ImPy–Fe complexes previously introduced (Chart [Fig chart1] and Table S2) could be included in this analysis. As detailed above, the investigated iron complexes comprise the five mononuclear (**M2**–**M5**) and the four dinuclear (**D6**–**D9**) species for which reliable XRD parameters are available. These experimental structures are represented as orange symbols in the correlation plots.

When distance-based descriptors are considered (Figure 1a and Figure 1b), the experimental data display a remarkably consistent behavior. All five mononuclear complexes exhibit average Fe–ligand distances fully compatible with HS configurations, in excellent agreement with available experimental magnetic susceptibility data for **M1,** ^57^ **M2,** ^10^ **M3,** ^10^ and **M5** ^58^ matching the high-spin Fe^2+^ ions (S = 2). In this representation, the experimental points fall unambiguously within the HS region defined by the computational library. Similarly, when projected onto the ζ–Δ radial correlation (Figure 1b), the experimental points remain fully consistent with the HS structural regime. These results confirm that bond-length-based descriptors reliably reflect the magnetic spin assignment in real systems.

A more complex picture emerges when angular descriptors are examined. In the Θ–Cl–Fe–Cl (Figure 1c) and Θ–Σ (Figure 1d) correlation plots, the five experimental mononuclear complexes no longer form a uniform group. Although all are magnetically classified as HS, their angular parameters span a broader region, partially overlapping with the LS-like domain identified in the computational dataset. In particular, several experimentally characterized HS complexes display Θ values closer to those associated with LS structures in the optimized models. These results suggest that angular distortions in experimental systems are not strictly dictated by spin state, often deviating from the idealized geometries predicted for isolated molecules. This divergence between radial and angular descriptors is particularly noteworthy; it reveals that while the spin state is reliably encoded in bond-length metrics, angular distortions are less constrained, exhibiting substantial variation even within a single spin manifold. As a consequence, spectroscopic observables are expected to respond selectively to different features of the coordination sphere, and their interpretation requires careful consideration of both structural effects and the inherent limitations of the computational framework employed.

The scarcity of integrated structural and spectroscopic data poses a major challenge. Typically, IR and UV–Vis signatures are reported without corresponding crystallographic evidence, or vice versa. As a result, **M1** and **M5** represent the only systems for which reliable XRD structures are coupled with experimental spectra, serving as the only verified reference points for our analysis. Among them, **M1** constitutes our primary reference point offering a particularly rich dataset, as a complete computational exploration of all relevant geometric isomers and spin states was performed. This allows for a direct comparison between experimental and simulated spectra (Figure 7); similar benchmarking data for **M5** can be found in the Supporting Information (Figure S7). These two complexes also provide the opportunity to illustrate the practical application of the spectroscopic workflow proposed in Section 4.1.4, demonstrating how complementary IR and UV–Vis information can be combined to progressively refine the structural interpretation and ultimately identify the most plausible structural model.

**Figure 7:**
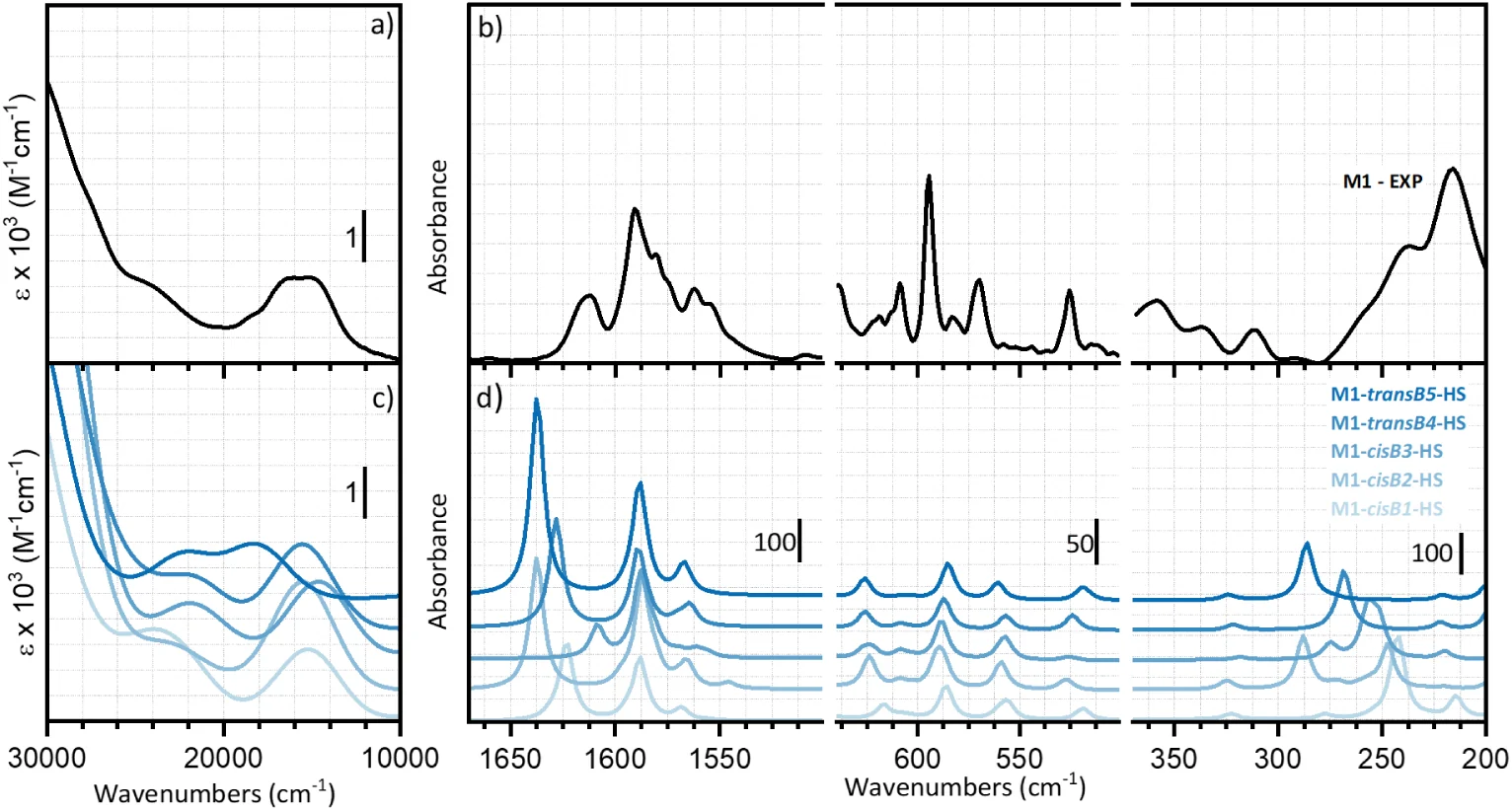
Comparison between experimental and simulated (a, c) UV–Vis and (b, d) IR spectra for **M1** considering all HS isomers. The UV–Vis spectra have been uniformly shifted by 5000 cm^–1^.

Following the proposed workflow, the interpretation starts from the ν­(Fe–Cl) region of the IR spectrum. For **M1**, the absence of the characteristic two-component ν­(Fe–Cl) pattern between 270 and 370 cm^–1^ immediately excludes the presence of pseudo-tetrahedral FeCl_2_ units, whereas the intense bands below 270 cm^–1^ are fully consistent with terminal chloride ligands bound to pseudo-octahedral HS Fe­(II) centers, in agreement with the crystallographic structure.

The analysis can then be refined by inspecting the ligand-centered ν­(C=N) region (Figure 7b). Although this spectral region is not included among the general diagnostic fingerprints because its behavior depends on the ligand framework, it provides useful additional information for **M1**. In particular, three HS isomers (***transB4***, ***transB5*** and ***cisB2***) exhibit an intense ν­(C=N) contribution above approximately 1610–1620 cm^–1^, whereas the experimental spectrum does not. This observation immediately reduces the number of plausible HS models from five to the two remaining *cis* arrangements (***cisB1*** and ***cisB3***). The final discrimination is then achieved by comparison of the complete experimental IR spectrum with the calculated vibrational fingerprints. While both *cis* isomers reproduce the main spectral features, the ***cisB1*** model provides the best agreement across all diagnostic regions, whereas ***cisB3*** still displays systematic discrepancies in both band positions and relative intensities. Thus, the IR spectrum alone allows the correct structural model to be recognized.

The final step consists in analyzing the MLCT region of the UV–Vis spectrum (Figure 7a). As previously noted, the low-energy absorption region is dominated by MLCT transitions involving donor orbitals localized on the FeCl_2_ fragment and ligand-centered acceptor orbitals. While the raw TD-DFT transition energies systematically underestimate the experimental excitations, consistent with recent benchmarks for iron complexes, the application of a uniform 5000 cm^–1^ energy shift allows the HS models to reproduce the experimental spectrum of **M1** with remarkable fidelity, capturing both the maximum position and the overall absorption profile.

The shifted UV–Vis spectra are fully consistent with the HS state independently established by XRD,^10,12,13,26,27,58^ and magnetic measurements.^10,57,58^ However, a comparison of the various HS isomers reveals that the electronic response is only moderately sensitive to specific coordination geometries. While fluctuations in band shape and intensity occur, several HS isomers exhibit qualitatively indistinguishable profiles once the empirical shift is applied. This limited structural sensitivity likely reflects the collective nature of the electronic transitions, which are governed by broad donor–acceptor orbital energetics rather than highly localized structural perturbations. Therefore, the UV–Vis spectrum provides an independent confirmation of the spin-state assignment suggested by the IR analysis, but does not by itself uniquely discriminate among the alternative structural models.

While the shifted TD-DFT spectra show excellent qualitative agreement with experimental data, the applied correction should be viewed as a system-specific calibration rather than a universally transferable constant. Instead, these calculations provide a robust interpretive framework for resolving the nature and relative energetic ordering of the electronic transitions.

Extending the same workflow to **M5** yields the same outcome (Figure S7). The ν­(Fe–Cl) region again identifies a pseudo-octahedral HS Fe­(II) environment, the N–Fe–N torsional region uniquely matches the ***cisB1*** **-HS** structural model, and the shifted UV–Vis spectrum consistently supports the same electronic configuration. Although **M5** bears a different ImPy ligand, the same sequence of spectroscopic interpretation correctly identifies the crystallographically determined structure.

Taken together, **M1** and **M5** demonstrate the practical applicability of the proposed spectroscopic workflow. Rather than relying on a single spectroscopic observable, the sequential interpretation of the ν­(Fe–Cl) region, the complete IR fingerprint and the MLCT transitions progressively reduces the number of plausible structural models, ultimately leading to the correct identification of the crystallographically determined structures. These two case studies therefore illustrate how the workflow proposed in Section 4.1.4 can be applied in practice for the spectroscopic interpretation of pseudo-octahedral ImPy–Fe­(II) complexes.

## 5 Conclusions

This work establishes a robust computational perspective on the structure–property relationships of iminopyridine Fe­(II) complexes across a chemically diverse structural library. By integrating an exhaustive isomer/spin-state exploration of the benchmark complex **M1** with a set of mononuclear and dinuclear iron complexes yet published, we identified descriptors that transcend variations in nuclearity and ligand design. These results provide critical clarity on the interpretive limits of spectroscopy, identifying which observables serve as reliable proxies for spin state versus those that resolve the intricacies of the local coordination sphere, thereby establishing the basis of a diagnostic spectroscopic framework for interpreting structurally flexible pseudo-octahedral ImPy–Fe­(II) complexes.

Our analysis demonstrates that distance-based descriptors (⟨*D*⟩ and ζ) serve as reliable and transferable markers of spin state across the entire dataset, remaining only marginally affected by isomerism or ligand variation. In contrast, angular descriptors exhibit significantly greater flexibility and broader overlap between LS and HS configurations, highlighting the increased structural adaptability of the HS manifold. Crucially, the vibrational response is found to be intimately coupled to the local coordination environment; IR spectroscopy, in particular, emerges as the most structurally diagnostic probe, capable of resolving not only the electronic spin-state but also distinguishing between closely related geometric isomers within a single spin manifold. On the other hand, the electronic response is governed by the energetic modulation of the metal-centered donor orbitals involved in MLCT transitions. Specifically, increasing the radial distortion, Δ, stabilizes these donor orbitals, thereby shifting the MLCT transitions to higher energies, while increasing the torsional distortion, Θ, exerts the opposite effect. Upon applying the empirical energy correction established for analogous iron complexes, the simulated UV–Vis spectra exhibit remarkable fidelity to the experimental visible absorption. While these electronic signatures are fully consistent with the HS structural regime, they remain intrinsically less sensitive than vibrational modes to subtle geometric differences. Consequently, UV–Vis spectroscopy serves primarily as a probe of the global electronic landscape rather than a high-resolution structural fingerprint.

More broadly, the present work establishes a diagnostic spectroscopic framework for interpreting structurally flexible pseudo-octahedral iminopyridine Fe­(II) complexes. Rather than providing a standalone predictive protocol for structural determination, the proposed methodology integrates experimentally accessible IR and UV–Vis observables with robust structure–property relationships to progressively constrain the range of plausible coordination environments and spin-state assignments. The applicability of this approach is demonstrated through the spectroscopic interpretation workflow developed in this work and illustrated using the experimentally characterized **M1** and **M5** benchmark complexes.

Within the family of iminopyridine Fe­(II) complexes investigated herein, this methodology provides a practical framework for the interpretation of spectroscopic data even in the absence of crystallographic information. It also establishes a rational basis for the structure-guide development of future ImPy–iron complexes for catalytic applications, including the (co)­polymerization of conjugated dienes to produce elastomers with a reduced carbon footprint.^8,12,18^

## Supplementary Material





## Notes

Cartesian coordinates of calculated structures (XYZ)
Additional energetic, structural, magnetic, vibrational, and electronic analyses, Principal Component Analysis (PCA), and the M5 case study (PDF)
