# Supplementary material for: Atomistic Origin of Structural, Vibrational, and Electronic Fingerprints in Iminopyridine Iron(II) Dichloride Complexes
Source: Inorg Chem. 2026 Sep 4;65(37):21615–28. doi: 10.1021/acs.inorgchem.6c02696 (PMC13599391; doi:10.1021/acs.inorgchem.6c02696)
Supplement: Supplementary file 1 [file ic6c02696_si_002.pdf]

SUPPORTING INFORMATIONS

Atomistic Origin of Structural, Vibrational and Electronic Fingerprints in Iminopyridine Iron(II) Dichloride Complexes

Anna Maria Fovanna[a], Giuseppe Leone[b], Marco Cavazzini[c], Laura Falivene[d,], Elena Groppo[a]*

[a] Department of Chemistry, NIS Interdepartmental Research Center and INSTM Reference Center, University of Torino, via G. Quarello 15/A, I-10135 Torino, Italy.

[b] CNR-Istituto di Scienze e Tecnologie Chimiche "Giulio Natta" (SCITEC), via A. Corti 12, I-20133 Milano, Italy.

[c] CNR-Istituto di Scienze e Tecnologie Chimiche "Giulio Natta" (SCITEC), via Golgi 19, I-20133 Milano, Italy.

[d] Department of Chemistry and Biology, University of Salerno, Via Ponte don Melillo, 24 - 84084 Fisciano (SA), Italy.

Corresponding Author

*lafalivene@unisa.it

Table of contents

S1. Energetic analysis and relative stability of M1 and D1 isomers

- Table S1. Relative Gibbs and electronic energies of **M1** and **D1** isomers.
- Figure S1. Intramolecular non-covalent interactions in **M1-*cisB1*-HS** and **M1-*cisB2*-HS**.

S2. Structural and magnetic properties of experimentally available and calculated complexes

- Table S2. Experimental structural descriptors and magnetic properties.
- Table S3. Structural descriptors of the calculated structures.
- Table S4. Comparison between optimized and experimental structure of **M1-*cisB1*-HS**.
- Figure S2. Correlation between Δ and ⟨D⟩.

S3. Principal Component Analysis (PCA)

- Table S5. PCA scores and cluster assignment.
- Figure S3. PCA score and loading plots.
- Table S6. Contributions of the structural descriptors to PC1 and PC2.

S4. Vibrational analysis

- Figure S4. Simulated IR spectra.
- Table S7. ν(C=N) analysis and fitted values.

S5. Electronic analysis

- Figure S5. Natural Transition Orbitals (NTOs).
- Figure S6. Donor and acceptor orbital energies.
- Table S8. MLCT analysis and fitted values.

S6. Experimental data in the context of the computational landscape: the M5 case study

- Figure S7. Comparison between experimental and simulated spectra for **M5**.

S1. Energetic analysis and relative stability of M1 & D1 isomers

Table S1. Gibbs free energies (ΔG) and Electronic energies (ΔE) of all optimized **M1** and **D1** isomers calculated at the B3LYP-D3(BJ)/def2-SVP level of theory with the SMD solvation model ($CHCl_3$). Relative energies are reported with respect to the lowest-energy structure in each set.

Part A: Relative energies of different isomers in the same spin state

a) M1 isomers - LS

Type of Ligand	Nuclearity	Isomers	Spin state	ΔG (eV)	ΔE (eV)
1	**M1**	cisB1	LS	**0.000**	**0.000**
1	**M1**	cisB2	LS	0.171	0.204
1	**M1**	cisB3	LS	0.433	0.464
1	**M1**	transB4	LS	0.518	0.537
1	**M1**	transB5	LS	0.370	0.482

b) M1 isomers – HS

Type of Ligand	Nuclearity	Isomers	Spin state	ΔG (eV)	ΔE (eV)
1	**M1**	cisB1	HS	**0.000**	**0.000**
1	**M1**	cisB2	HS	0.103	0.096
1	**M1**	cisB3	HS	0.357	0.385
1	**M1**	transB4	HS	0.326	0.285
1	**M1**	transB5	HS	0.183	0.211

c) D1 isomers – *LS*LS

Type of Ligand	Nuclearity	Isomers	Spin state	ΔG (eV)	ΔE (eV)
1	**D1**	cisB1	***LS***LS	**0.000**	**0.000**
1	**D1**	cisB2	***LS***LS	0.269	0.223
1	**D1**	cisB3	***LS***LS	0.662	0.594

d) D1 isomers -*LS*HS

Type of Ligand	Nuclearity	Isomers	Spin state	ΔG (eV)	ΔE (eV)
1	**D1**	cisB1	***LS***HS	**0.000**	**0.000**
1	**D1**	cisB2	***LS***HS	0.169	0.059
1	**D1**	cisB3	***LS***HS	0.556	0.476

e) D1 isomers – *HS*HS

Type of Ligand	Nuclearity	Isomers	Spin state	ΔG (eV)	ΔE (eV)
1	**D1**	cisB1	***HS***HS	**0.000**	**0.000**
1	**D1**	cisB2	***HS***HS	0.057	0.004
1	**D1**	cisB3	***HS***HS	0.334	0.286

Part B: Relative energies of different spin states for the same isomer

a) M1-*cisB1* – Spin State comparison

Type of Ligand	Nuclearity	Isomers	Spin state	ΔG (eV)	ΔE (eV)
1	**M1**	cisB1	HS	**0.000**	**0.000**
1	**M1**	cisB1	LS	0.767	0.490

b) D1-*cisB1* – Spin State comparison

Type of Ligand	Nuclearity	Isomers	Spin state	ΔG (eV)	ΔE (eV)
1	**D1**	cisB1	***HS***HS	**0.000**	**0.000**
1	**D1**	cisB1	***LS***HS	0.546	0.386
1	**D1**	cisB1	***LS***LS	3.330	3.031

Investigation of the Optimized Geometries of M1 Isomers

The relative stability of the **M1** isomers can be rationalized by considering both intramolecular non-covalent interactions and the coordination geometry. In the lowest-energy isomer, **M1-*cisB1*-HS**, which is fully consistent with the experimentally determined molecular structure, the two ligands adopt a favorable arrangement that enables efficient face-to-face π–π interactions between the pyridine ring of each ligand and the phenyl group attached to the iminic nitrogen of the opposite ligand (**Figure S1a**). These attractive interactions contribute to the stabilization of the complex. Moreover, **M1-*cisB1*-HS** displays a relatively regular octahedral coordination environment (**Table S3**), with low angular distortion parameters, suggesting a favorable balance between ligand

arrangement and metal coordination. In the **M1-*cisB2*-HS** isomer, the different mutual orientation of the ligands no longer allows these π–π contacts. Instead, only weaker C–H···π interactions can be established (**Figure S1b**), resulting in a less favorable stabilization. Although this isomer maintains a coordination geometry comparable to that of **M1-*cisB1*-HS**, the loss of more favorable interligand interactions results in a slightly reduced stability. In contrast, **M1-*cisB3*-HS** combines the absence of significant stabilizing interligand interactions with a more distorted coordination environment, as indicated by the larger angular distortion parameters, leading to the lowest relative stability among the investigated **M1** isomers. The trans isomers exhibit intermediate stability, reflecting a different balance between ligand arrangement, coordination geometry, and weak intramolecular interactions.

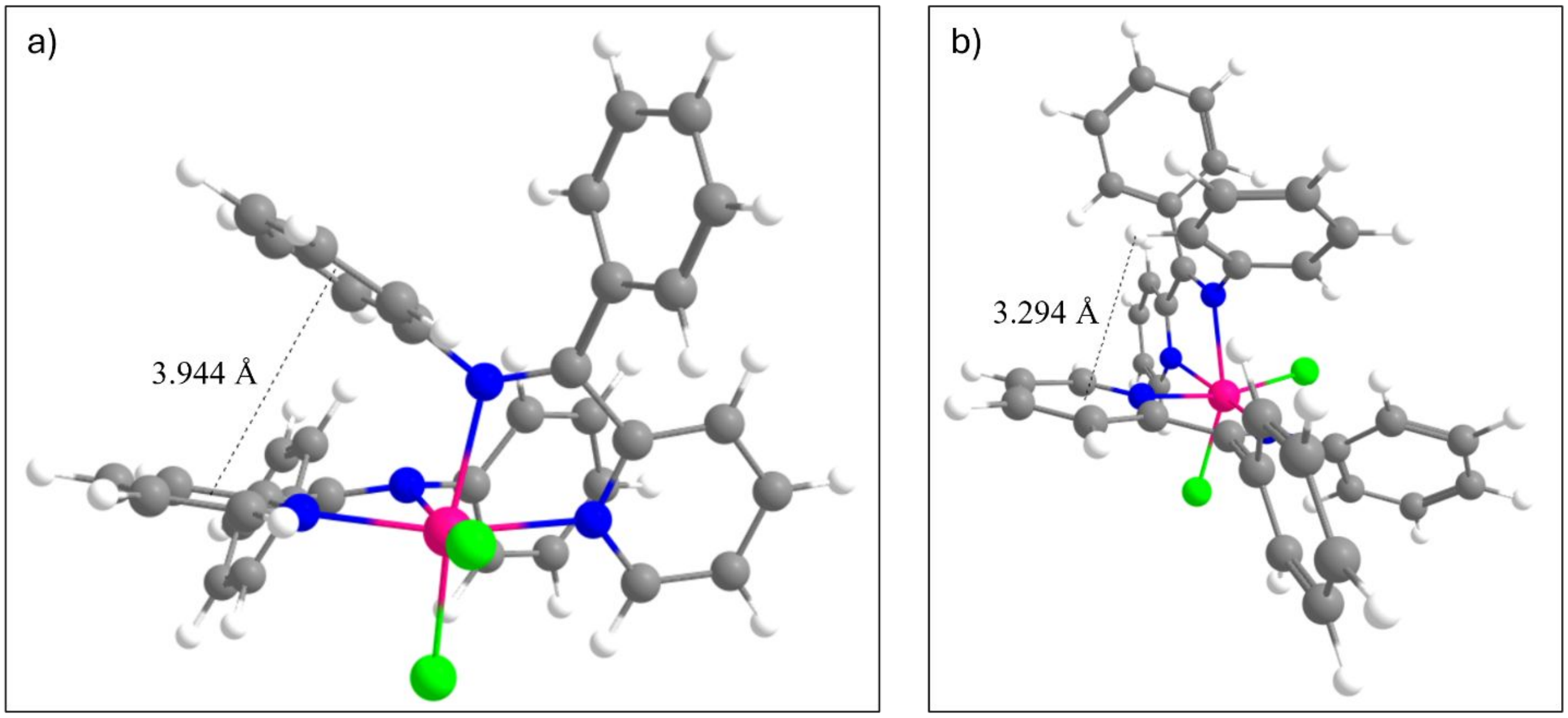

Figure S1. Optimized geometries of the **M1-*cisB1*-HS** (a) and **M1-*cisB2*-HS** (b) isomers highlighting the principal intramolecular non-covalent interactions responsible for their relative stabilization. Selected intermolecular distances are reported in Å.

S2. Structural and magnetic properties for experimentally available and calculated complexes

Table S2. Structural descriptors and available experimental magnetic properties of the pseudo-octahedral ImPy–Fe(II) complexes reported in the literature and used as experimental references in this work. The effective magnetic moments (μ_{eff}) are taken from the corresponding literature sources or determined in the present work for **M1** using the Evans method.

	Type of Ligand	Nuclearity	Isomers	<D>(Å)	ζ (Å)	Δ	Σ (°)	Θ (°)	Cl-Fe-Cl (°)	μ_{eff} (BM)	Ref.
1	1	**M1-exp**	cisB1	2.281	0.491	0.002	85.39	230.04	98.86	4.50	d
2	2	**M2-exp**	cisB1	2.298	0.404	0.001	95.83	342.52	105.58	6.34	e
3	3	**M3-exp**	cisB1	2.298	0.412	0.001	96.04	352.85	95.16	6.37	e
4	4	**M4-exp**	cisB1	2.311	0.415	0.001	90.91	279.78	98.24	-	
5	5	**M5-exp[a]**	cisB1	2.285	0.505	0.002	91.15	238.02	100.50	-	
6	5	**M5-exp[b]**	cisB1	2.280	0.496	0.002	89.14	226.03	97.99	-	
7	5	**M5-exp[c]**	cisB1	2.288	0.514	0.002	79.02	211.97	96.52	5.49	c
8	6	**D6-exp**	cisB1	2.259	0.733	0.003	72.53	257.83	88.68	5.30*	e
9	7	**D7-exp**	cisB1	2.295	0.560	0.002	94.32	365.07	86.09	5.10*	e
10	8	**D8-exp**	cisB1	2.303	0.743	0.003	76.10	243.22	85.04	-	
11	9	**D9-exp**	cisB1	2.269	0.823	0.004	61.15	201.54	87.64	-	

*The magnetic moment reported is given per iron atom.

Ref. a: Chaggar, R. K.; Fawcett, J.; Solan, G. A. Dichlorobis(2-Formyl-pyridine-anil)-iron(II) Aceto-nitrile Solvate. *Acta Cryst E* **2003**, *59* (7), m462–m463. https://doi.org/10.1107/S1600536803012467.

Ref. b: Ricci, G.; Leone, G.; Zanchin, G.; Palucci, B.; Boccia, A. C.; Sommazzi, A.; Masi, F.; Zacchini, S.; Guelfi, M.; Pampaloni, G. Highly Stereoregular 1,3-Butadiene and Isoprene Polymers through Monoalkyl- *N*- Aryl-Substituted Iminopyridine Iron Complex-Based Catalysts: Synthesis and Characterization. *Macromolecules* **2021**, *54* (21), 9947–9959. https://doi.org/10.1021/acs.macromol.1c01291.

Ref c: Tsaulwayo, N.; Kumah, R. T.; Ojwach, S. O. Synthesis and Structural Elucidation of (Pyridyl)Imine Fe(II) Complexes and Their Applications as Catalysts in Transfer Hydrogenation of Ketones. *Polyhedron* **2021**, *197*, 115034. https://doi.org/10.1016/j.poly.2021.115034.

Ref d: Measurements Performed Using the Evans Method at Varying Temperatures (CD_2Cl_2/CH_2Cl_2 10:1 v/v, c = 0.01679 mol·L^{-1}) Confirm the Presence of a Paramagnetic Species in Solution with Meff = 4.41–4.58 BM in the Range of Temperature from 293 to 251 K.

Ref e: Hashmi, O. H.; Caulier, R.; Capet, F.; Mentré, O.; Minaud, C.; Meunier, J.-F.; Del Rosal, I.; Dinoi, C.; Visseaux, M.; Champouret, Y. Mono- to Tetra-Nuclear Iron Dichloride Complexes Bearing N -(Fluoro)-Aryl-Substituted Iminopyridine Ligands: Synthesis, Structures, Magnetic

Properties, and Polymerization of Isoprene. Inorg. Chem. 2025, 64 (29), 14836–14852. https://doi.org/10.1021/acs.inorgchem.5c01030.

Table S3. Structural descriptors and magnetic properties for all 37 optimized structures.

	Type of Ligand	Nuclearity	Isomers	Spin state	<D>(Å)	ζ (Å)	Δ	Σ (°)	Θ (°)	Cl-Fe-Cl (°)
1	1	**M1**	cisB1	LS	2.099	1.088	0.008	64.52	180.23	94.22
2	1	**M1**	cisB2	LS	2.103	1.054	0.008	61.84	193.00	97.00
3	1	**M1**	cisB3	LS	2.105	1.054	0.008	67.20	195.88	96.18
4	1	**M1**	transB4	LS	2.121	0.974	0.007	81.18	240.71	177.96
5	1	**M1**	transB5	LS	2.122	1.000	0.007	81.67	226.50	180.00
6	1	**M1**	cisB1	HS	2.284	0.467	0.002	98.73	282.86	110.18
7	1	**M1**	cisB2	HS	2.287	0.396	0.001	97.45	286.68	108.85
8	1	**M1**	cisB3	HS	2.291	0.421	0.001	115.55	315.38	109.26
9	1	**M1**	transB4	HS	2.298	0.472	0.001	103.18	322.32	176.64
10	1	**M1**	transB5	HS	2.303	0.576	0.003	114.74	282.34	180.00
11	1	**D1**	cisB1	***LS***LS	2.105	1.154	0.009	62.03	171.13	90.09
12	1	**D1**	cisB1	***LS***HS	2.107	1.156	0.009	64.60	181.00	93.20
13	1	**D1**	cisB1	***HS***HS	2.281	0.799	0.004	82.15	260.50	102.64
14	1	**D1**	cisB2	***LS***LS	2.117	1.126	0.009	57.63	170.66	85.50
15	1	**D1**	cisB2	***LS***HS	2.114	1.117	0.009	54.06	170.60	89.82
16	1	**D1**	cisB2	***HS***HS	2.282	0.749	0.003	71.54	221.67	94.10
17	1	**D1**	cisB3	***LS***LS	2.120	1.069	0.008	63.64	174.77	86.07
18	1	**D1**	cisB3	***LS***HS	2.121	1.082	0.008	59.99	168.01	88.17
19	1	**D1**	cisB3	***HS***HS	2.299	0.719	0.003	79.76	245.46	89.18
20	2	**M2**	cisB1	LS	2.107	0.968	0.007	53.69	190.70	96.45
21	2	**M2**	cisB1	HS	2.315	0.259	0.000	113.40	416.96	116.25
22	3	**M3**	cisB1	LS	2.103	0.962	0.007	62.33	173.80	92.27
23	3	**M3**	cisB1	HS	2.305	0.349	0.001	97.84	280.93	106.10
24	4	**M4**	cisB1	LS	2.104	0.979	0.007	61.85	172.51	92.07
25	4	**M4**	cisB1	HS	2.303	0.364	0.001	94.48	273.50	105.95
26	5	**M5**	cisB1	LS	2.106	1.010	0.007	51.20	181.05	95.59
27	5	**M5**	cisB1	HS	2.300	0.398	0.001	95.02	245.34	103.94
28	6	**D6**	cisB1	***LS***LS	2.108	1.051	0.008	53.08	179.66	91.11
29	6	**D6**	cisB1	***LS***HS	2.110	1.091	0.008	57.04	189.20	93.73
30	6	**D6**	cisB1	***HS***HS	2.291	0.673	0.003	82.37	280.40	101.69
31	8	**D8**	cisB1	***LS***LS	2.121	1.132	0.009	61.23	176.42	91.25
32	8	**D8**	cisB1	***LS***HS	2.119	1.125	0.009	61.02	176.56	91.46
33	8	**D8**	cisB1	***HS***LS	2.287	0.840	0.004	78.10	243.47	93.55

34	8	**D8**	cisB1	***HS*HS**	2.288	0.811	0.004	78.64	241.39	94.68
35	9	**D9**	cisB1	***LS*LS**	2.116	1.117	0.009	54.81	186.41	92.86
36	9	**D9**	cisB1	***LS*HS**	2.120	1.101	0.008	58.68	196.91	92.41
37	9	**D9**	cisB1	***HS*HS**	2.297	0.713	0.003	79.83	283.92	99.47

Table S4. Key structural parameters for optimized **M1-*cisB1*-HS** and the experimental XRD structure. Distances are in Å, and angles in °.

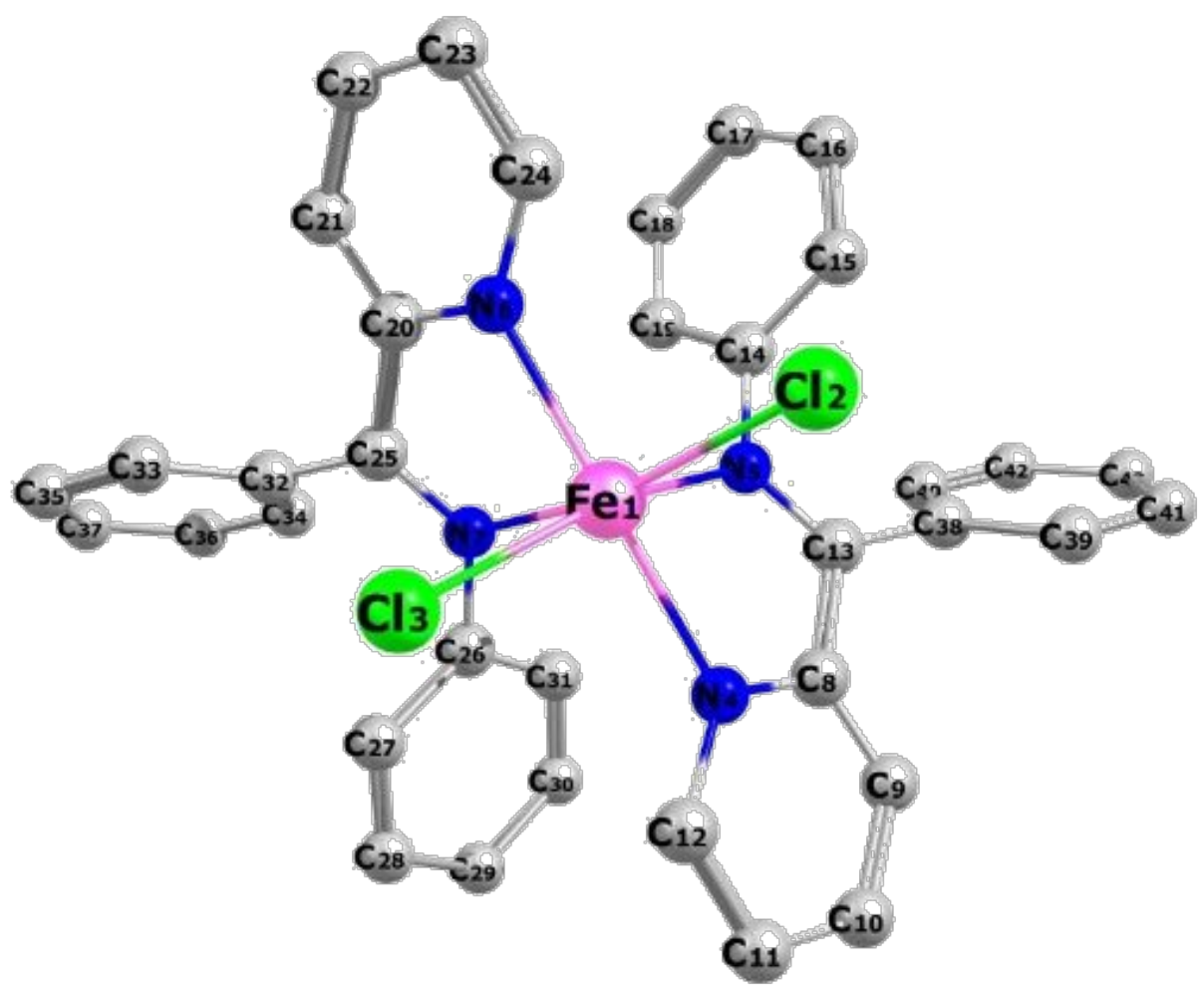

	XRD	DFT (HS)	Δcalc-exp
**Fe1-Cl2**	2.394	2.400	0.006
**Fe1-Cl3**	2.412	2.400	-0.012
**Fe1-N4**	2.169	2.181	0.012
**Fe1-N5**	2.274	2.269	-0.004
**Fe1-N6**	2.172	2.181	0.009
**Fe1-N7**	2.263	2.269	0.007
**N4-C8**	1.350	1.349	-0.002
**N5-C13**	1.277	1.286	0.009
**C8-C13**	1.490	1.489	-0.001
**N6-C20**	1.355	1.349	-0.006
**N7-C25**	1.281	1.286	0.005
**C20-C25**	1.482	1.489	0.008
**Cl2-Fe1-Cl3**	98.86	110.18	11.31
**N4-Fe1-N5**	72.89	73.58	0.69
**N6-Fe1-N7**	73.00	73.58	0.58
**Fe1-N4-C8**	118.00	115.88	-2.11
**Fe1-N5-C13**	116.73	113.32	-3.41
**Fe1-N6-C20**	117.84	115.89	-1.95
**Fe1-N7-C25**	116.86	113.32	-3.54

**N4-C8-C13**	115.41	115.96	0.56
**N5-C13-C8**	115.99	115.96	-0.03
**N6-C20-C25**	115.3	115.96	0.67
**N7-C25-C20**	116.71	116.16	-0.55

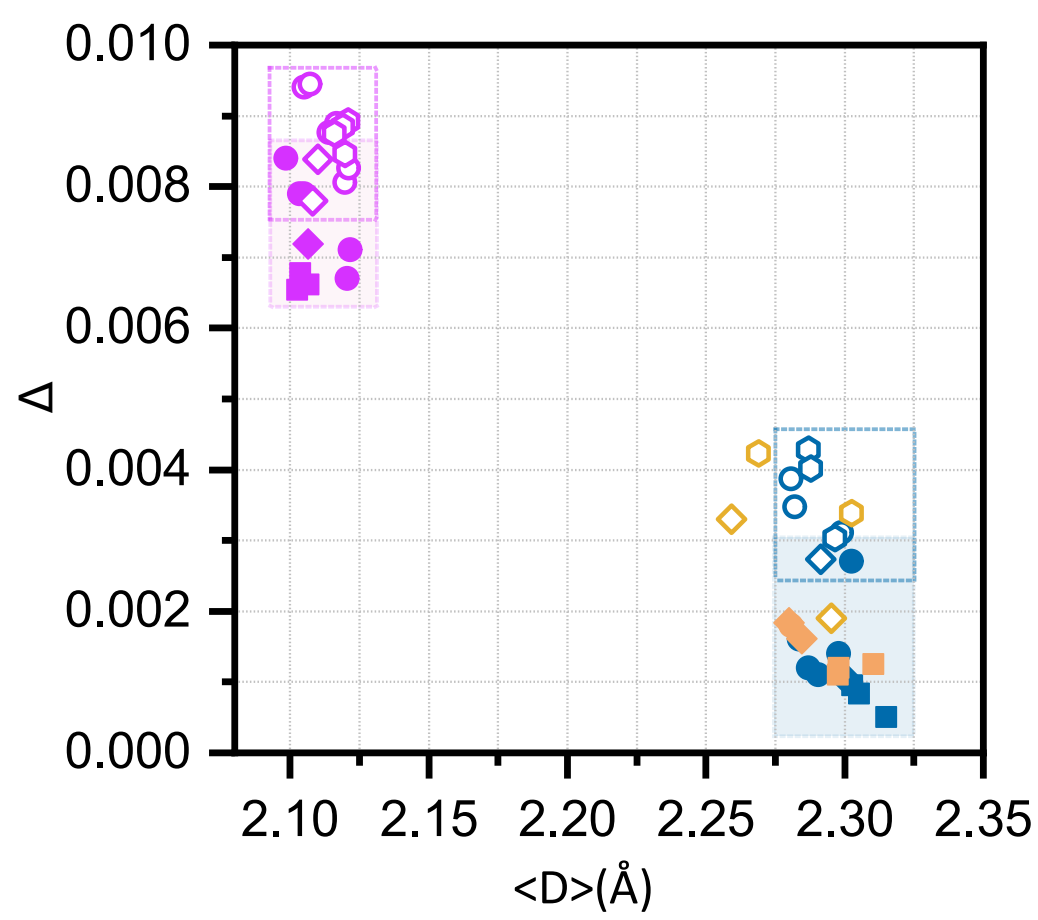

Figure S2. Correlation between the normalized radial distortion parameter Δ and the average Fe–ligand bond length ⟨D⟩ for all optimized pseudo-octahedral ImPy–Fe(II) complexes. The monotonic relationship confirms the internal consistency between the two distance-based descriptors discussed in the main text.

S3. PCA Analysis

Table S5. Principal component coordinates (PC1 and PC2) obtained from the PCA analysis for all calculated structures together with the corresponding cluster assignment.

Label	Nuclearity	Isomers	Spin state	PC1	PC2	Cluster_ID	Cluster
**M1-LS**	**M1**	cisB1	LS	-1.665	0.140	1	LS-cis
**M1-LS**	**M1**	cisB2	LS	-1.515	0.169	1	LS-cis
**M1-LS**	**M1**	cisB3	LS	-1.347	0.154	1	LS-cis
**M1-LS**	**M1**	transB4	LS	0.459	0.287	2	Trans
**M1-LS**	**M1**	transB5	LS	0.332	2.969	2	Trans
**M1-HS**	**M1**	cisB1	HS	0.228	-0.431	0	HS-cis
**M1-HS**	**M1**	cisB2	HS	0.241	-0.555	0	HS-cis
**M1-HS**	**M1**	cisB3	HS	0.031	-0.480	0	HS-cis
**M1-HS**	**M1**	transB4	HS	3.482	0.179	2	Trans
**M1-HS**	**M1**	transB5	HS	0.332	0.021	2	Trans
**D1-LS**	**D1**	cisB1	LS	-1.930	0.023	1	LS-cis
**D1-LS**	**D1**	cisB1	LS	-1.738	0.127	1	LS-cis
**D1-HS**	**D1**	cisB1	HS	0.994	-0.463	0	HS-cis
**D1-LS**	**D1**	cisB2	LS	-1.989	-0.218	1	LS-cis
**D1-LS**	**D1**	cisB2	LS	-2.036	-0.083	1	LS-cis
**D1-HS**	**D1**	cisB2	HS	0.387	-0.817	0	HS-cis
**D1-LS**	**D1**	cisB3	LS	-0.168	-0.234	1	LS-cis
**D1-LS**	**D1**	cisB3	LS	-1.825	-0.166	1	LS-cis
**D1-HS**	**D1**	cisB3	HS	0.895	-1.057	0	HS-cis
**M2-LS**	**M2**	cisB1	LS	-1.591	0.037	1	LS-cis
**M2-HS**	**M2**	cisB1	HS	0.439	-0.548	0	HS-cis
**M3-LS**	**M3**	cisB1	LS	-0.156	-0.049	1	LS-cis
**M3-HS**	**M3**	cisB1	HS	0.251	-0.748	0	HS-cis
**M4-LS**	**M4**	cisB1	LS	-1.610	-0.046	1	LS-cis
**M4-HS**	**M4**	cisB1	HS	2.319	-0.741	0	HS-cis
**M5-LS**	**M5**	cisB1	LS	-1.820	0.038	1	LS-cis
**M5-HS**	**M5**	cisB1	HS	1.998	-0.747	0	HS-cis
**D6-LS**	**D6**	cisB1	LS	-1.890	-0.080	1	LS-cis
**D6-LS**	**D6**	cisB1	LS	-1.736	0.044	1	LS-cis
**D6-HS**	**D6**	cisB1	HS	0.143	-0.652	0	HS-cis
**D8-LS**	**D8**	cisB1	LS	-1.775	-0.020	1	LS-cis
**D8-LS**	**D8**	cisB1	LS	-1.776	-0.012	1	LS-cis
**D8-HS**	**D8**	cisB1	HS	0.610	-0.770	0	HS-cis
**D8-HS**	**D8**	cisB1	HS	0.672	-0.754	0	HS-cis
**D9-LS**	**D9**	cisB1	LS	-1.841	0.006	1	LS-cis
**D9-LS**	**D9**	cisB1	LS	-0.161	-0.027	1	LS-cis
**D9-HS**	**D9**	cisB1	HS	1.330	-0.729	0	HS-cis

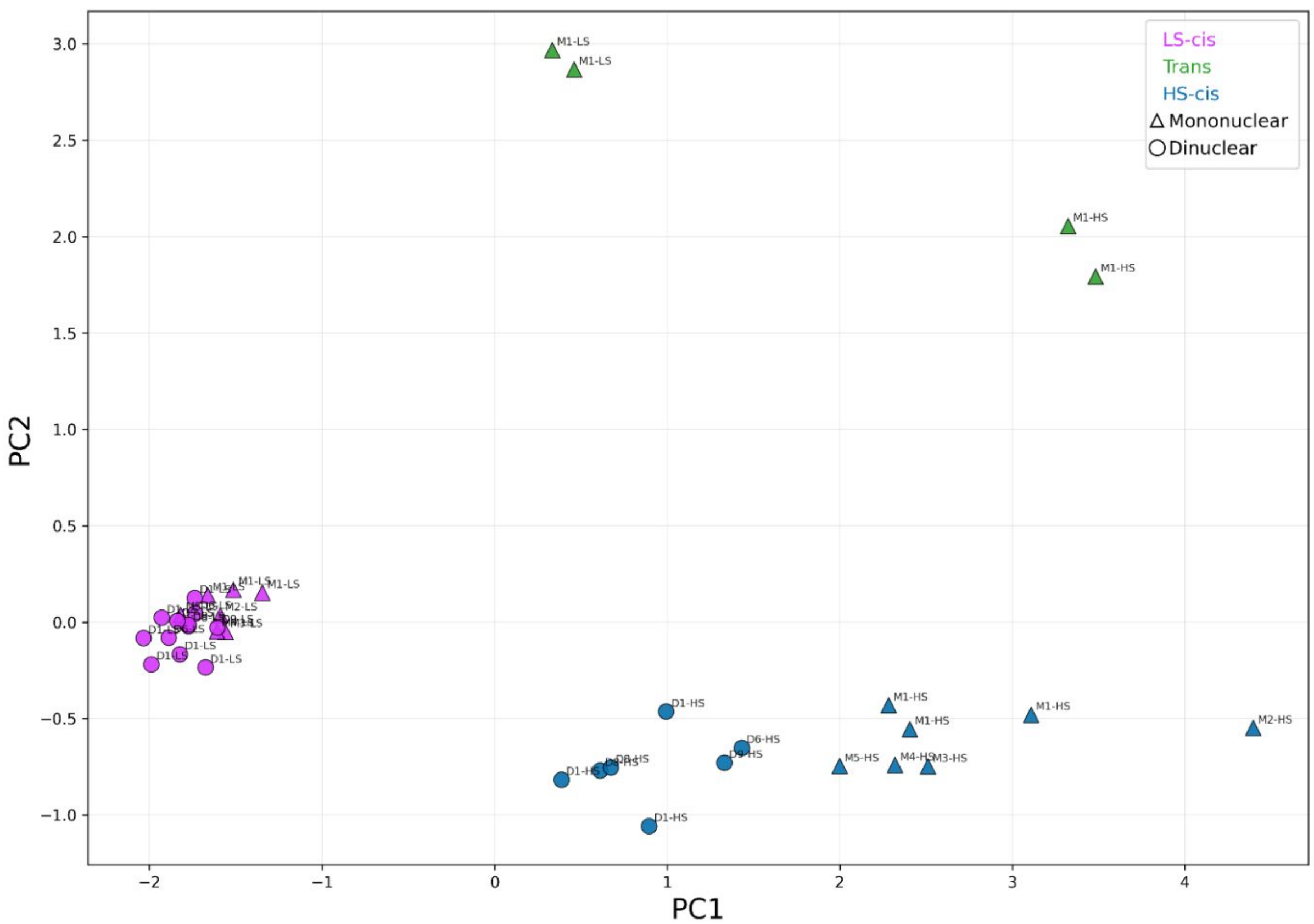

Figure S3. Principal Component Analysis of the complete structural descriptor dataset. Score plot showing the distribution of the calculated structures along the first two principal components.

Table S6. Relative contribution of each structural descriptor to the first two principal components obtained from the PCA analysis.

	**PC1 (%)**	**PC2 (%)**
**<D>(Å)**	21.3	11.2
**ζ (Å)**	23.3	5.2
**Σ (°)**	24.4	0.6
**Θ (°)**	23.6	0.2
**Cl-Fe-Cl (°)**	7.4	82.8

S4. Vibrational analysis

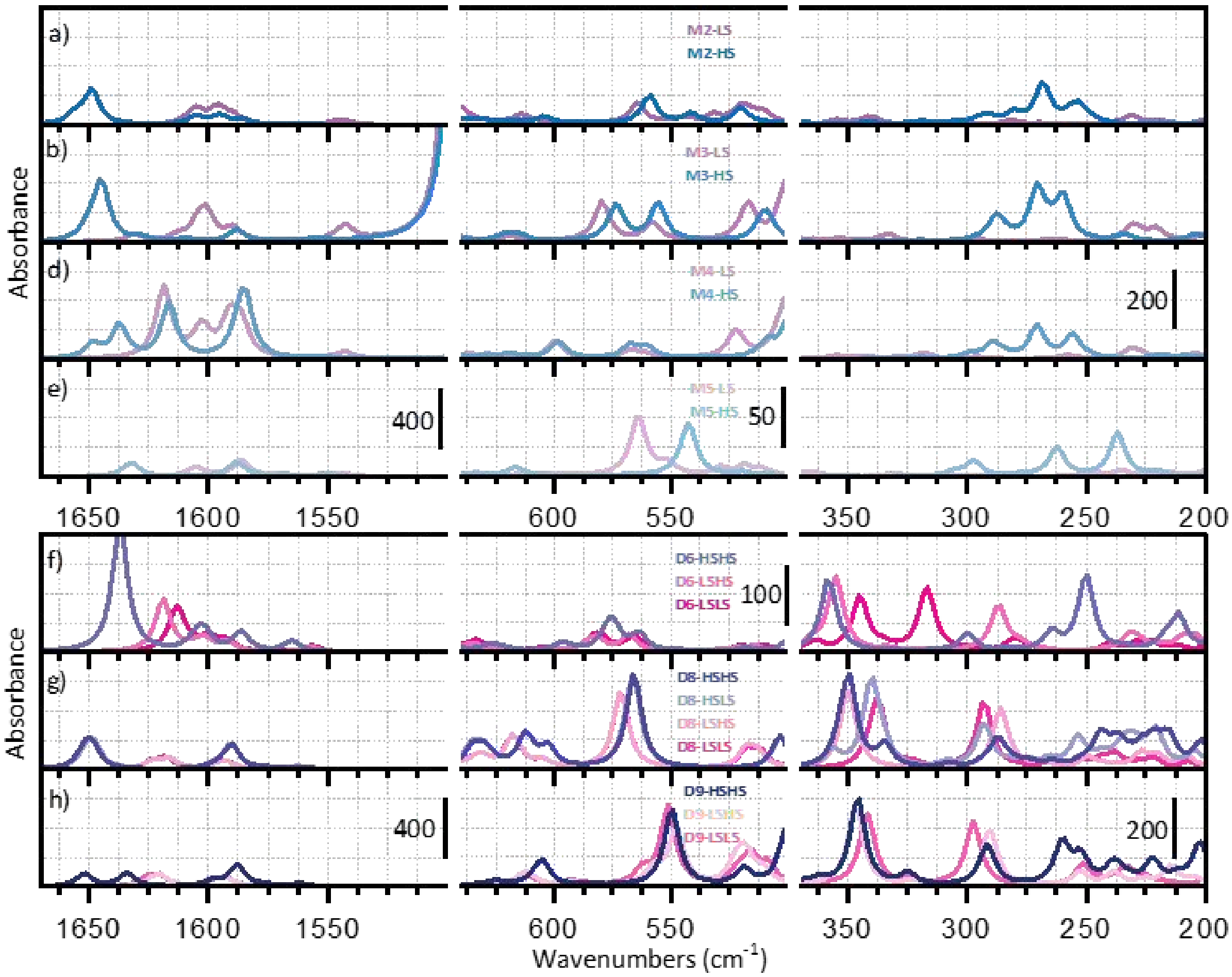

Figure S4. Simulated IR spectra for mononuclear complexes **M2–M5** (a–e) and dinuclear complexes **D6**, **D8** and **D9** (f–h), reported for both low-spin (LS) and high-spin (HS) electronic configurations. The spectra are shown in the same three diagnostic regions identified for the reference systems **M1** and **D1**. While the overall spectral profiles are strongly affected by the nature of the iminopyridine ligand, consistent qualitative trends with respect to spin state are preserved across the entire series, confirming the transferability of the vibrational signatures identified for **M1** and **D1**.

Table S7. Calculated ν(C=N) stretching wavenumbers together with the corresponding structural descriptors (Σ, Θ and buried volumes) used for the multivariate analysis. The last column reports the values predicted by the fitted model.

**Type of Ligand**	**Nuclearity**	**Isomers**	**Spin state**	**ν(C=N)$_{DFT}$ (cm^{-1})**	**Σ (°)**	**Θ (°)**	**%V$_{Bur}$(r1)**	**%V$_{Bur}$(r2)**	**ν(C=N)$_{fitted}$ (cm^{-1}) (%V$_{Bur}$, %V$_{DBur}$ Θ, Σ)**
1	**M1**	cisB1	LS	1592	64.52	180.23	70.48	60.55	1604
1	**M1**	cisB2	LS	1598	61.84	193.00	70.41	59.07	1597
1	**M1**	cisB3	LS	1593	67.20	195.88	71.35	59.6	1591
1	**M1**	transB4	LS	1593	81.18	240.71	68.27	56.86	1595
1	**M1**	transB5	LS	1602	81.67	226.50	69.06	59.04	1603
1	**M1**	cisB1	HS	1630	98.73	282.86	64.21	57.97	1636
1	**M1**	cisB2	HS	1637	97.45	286.68	63.62	56.11	1627
1	**M1**	cisB3	HS	1609	115.55	315.38	65.03	56.72	1615
1	**M1**	transB4	HS	1629	103.18	322.32	63.7	56.08	1631
1	**M1**	transB5	HS	1637	114.74	282.34	64.84	58.08	1620
1	**D1**	cisB1	***LS***LS	1594	62.03	171.13	70.77	60.7	1602
1	**D1**	cisB1	***LS***HS	1593	64.60	181.00	70.63	60.73	1605
1	**D1**	cisB1	***HS***HS	1616	82.15	260.50	66.21	58.4	1629
1	**D1**	cisB2	***LS***LS	1594	57.63	170.66	69.74	58.64	1596
1	**D1**	cisB2	***LS***HS	1594	54.06	170.60	70.04	58.9	1598
1	**D1**	cisB2	***HS***HS	1627	71.54	221.67	65.45	57.13	1622
1	**D1**	cisB3	***LS***LS	1600	63.64	174.77	69.55	58.99	1598
1	**D1**	cisB3	***LS***HS	1601	59.99	168.01	69.61	59.07	1599
1	**D1**	cisB3	***HS***HS	1607	79.76	245.46	64.68	56.6	1624
2	**M2**	cisB1	LS	1610	53.69	190.70	68.69	58.72	1613
2	**M2**	cisB1	HS	1656	113.40	416.96	61.29	54.89	1658
3	**M3**	cisB1	LS	1631	62.33	173.80	71.65	64.54	1629
3	**M3**	cisB1	HS	1650	97.84	280.93	62.75	59.22	1658
4	**M4**	cisB1	LS	1619	61.85	172.51	71.6	64.25	1627
4	**M4**	cisB1	HS	1648	94.48	273.50	63.37	59.39	1655
5	**M5**	cisB1	LS	1606	51.20	181.05	68.85	58.4	1608
5	**M5**	cisB1	HS	1634	95.02	245.34	59.96	53.87	1628
6	**D6**	cisB1	***LS***LS	1616	53.08	179.66	69.55	60.06	1615
6	**D6**	cisB1	***LS***HS	1623	57.04	189.20	69.75	60.29	1615
6	**D6**	cisB1	***HS***HS	1637	82.37	280.40	64.35	57.56	1642
8	**D8**	cisB1	***LS***LS	1624	61.23	176.42	71.54	62.76	1616
8	**D8**	cisB1	***LS***HS	1624	61.02	176.56	71.54	62.79	1616
8	**D8**	cisB1	***HS***HS	1652	78.64	241.39	66.68	60.29	1639
9	**D9**	cisB1	***LS***LS	1626	54.81	186.41	69.63	60.64	1620
9	**D9**	cisB1	***LS***HS	1623	58.68	196.91	70.18	60.09	1611
9	**D9**	cisB1	***HS***HS	1652	79.83	283.92	65.04	57.1	1635

S5. Electronic analysis

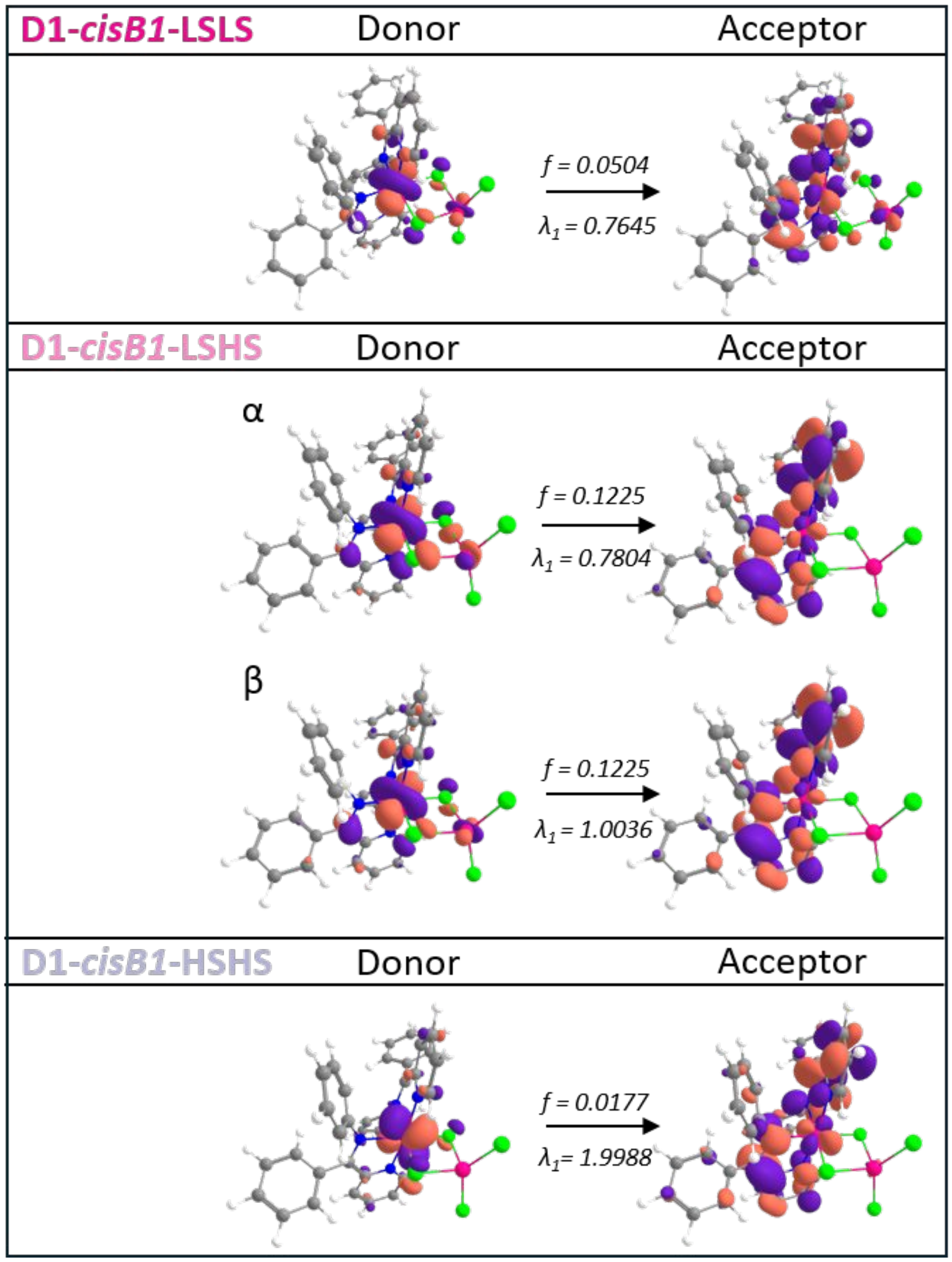

Figure S5. NTOs for **D1-*cisB1*-LSLS** (excited state 18), **D1-*cisB1*-LSHS** (excited state 24), and **D1-*cisB1*-HSHS** (excited state 9), together with the corresponding oscillator strengths (*f*) and dominant NTO pair weights (λ_1).

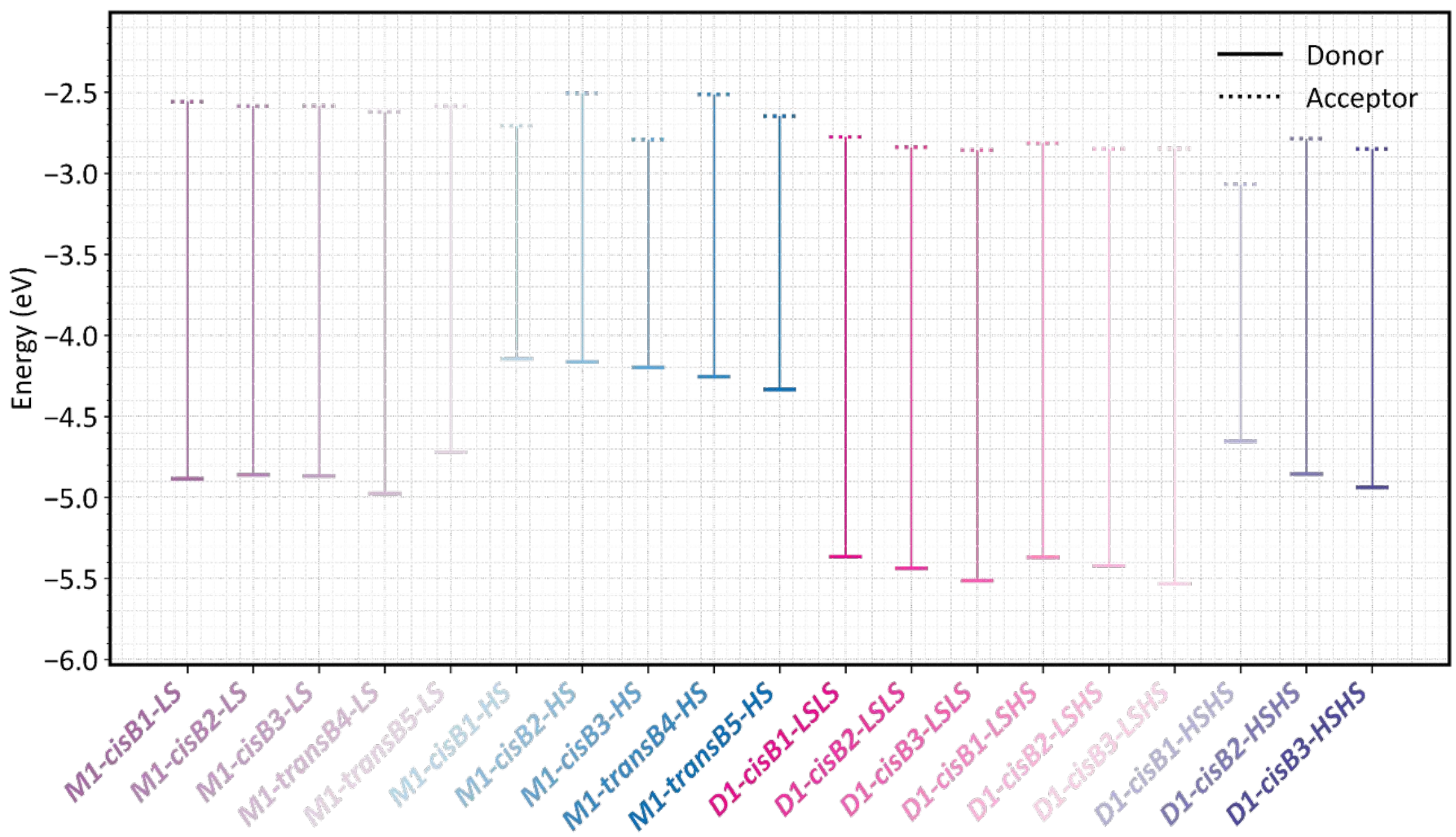

Figure S6. Energies of donor and acceptor orbitals involved in the MLCT transition for **M1** and **D1** species.

Table S8. Calculated MLCT transition energies together with the corresponding structural descriptors (Δ and Fe APT charges) employed in the multivariate analysis. The last column reports the MLCT energies predicted by the fitted model.

Type of Ligand	Nuclearity	Isomers	Spin state	$MLCT_{DFT}$ (cm^{-1})	Δ	Fe APT charges	$MLCT_{fitted}$ (cm^{-1}) (Δ, Fe APT charges)
1	**M1**	cisB1	LS	15431	0.0084	-0.0575	16717
1	**M1**	cisB2	LS	15380	0.0079	0.0378	16233
1	**M1**	cisB3	LS	15180	0.0079	0.0607	16292
1	**M1**	transB4	LS	15658	0.0067	-0.0045	14365
1	**M1**	transB5	LS	14217	0.0071	0.0573	15112
1	**M1**	cisB1	HS	10187	0.0016	1.3042	10300
1	**M1**	cisB2	HS	10521	0.0012	1.4326	10048
1	**M1**	cisB3	HS	9417	0.0011	1.4168	9861
1	**M1**	transB4	HS	10547	0.0014	1.5543	10658
1	**M1**	transB5	HS	13134	0.0027	1.5268	12490
1	**D1**	cisB1	***LS***LS	17817	0.0094	0.1567	18739
1	**D1**	cisB1	***LS***HS	17451	0.0094	0.1722	18842
1	**D1**	cisB1	***HS***HS	12350	0.0039	1.5169	14174
1	**D1**	cisB2	***LS***LS	17887	0.0089	0.2540	18250
1	**D1**	cisB2	***LS***HS	17578	0.0088	0.2365	18017
1	**D1**	cisB2	***HS***HS	13733	0.0035	1.5906	13780
1	**D1**	cisB3	***LS***LS	17271	0.0081	0.2817	17088
1	**D1**	cisB3	***LS***HS	17225	0.0083	0.3006	17448
1	**D1**	cisB3	***HS***HS	13813	0.0031	1.5957	13263
2	**M2**	cisB1	LS	14691	0.0066	0.1558	14656
2	**M2**	cisB1	HS	8925	0.0005	1.3978	8931
3	**M3**	cisB1	LS	14950	0.0065	0.0903	14379
3	**M3**	cisB1	HS	8965	0.0008	1.3508	9295
4	**M4**	cisB1	LS	15131	0.0068	0.0931	14720
4	**M4**	cisB1	HS	9670	0.0010	1.3602	9501
5	**M5**	cisB1	LS	15208	0.0072	0.1541	15491
5	**M5**	cisB1	HS	9946	0.0011	1.2810	9461
6	**D6**	cisB1	***LS***LS	17919	0.0078	0.2527	16640
6	**D6**	cisB1	***LS***HS	17987	0.0084	0.2479	17487
6	**D6**	cisB1	***HS***HS	13104	0.0027	1.5770	12666
8	**D8**	cisB1	***LS***LS	20093	0.0089	0.3771	18615
8	**D8**	cisB1	***LS***HS	18929	0.0088	0.3601	18454
8	**D8**	cisB1	***HS***HS	14993	0.0040	1.5158	14385
9	**D9**	cisB1	***LS***LS	19442	0.0088	0.3617	18320
9	**D9**	cisB1	***LS***HS	19249	0.0085	0.3582	17887
9	**D9**	cisB1	***HS***HS	11563	0.0030	1.5354	12996

S6. Experimental data in the context of the computational landscape: the M5 case study

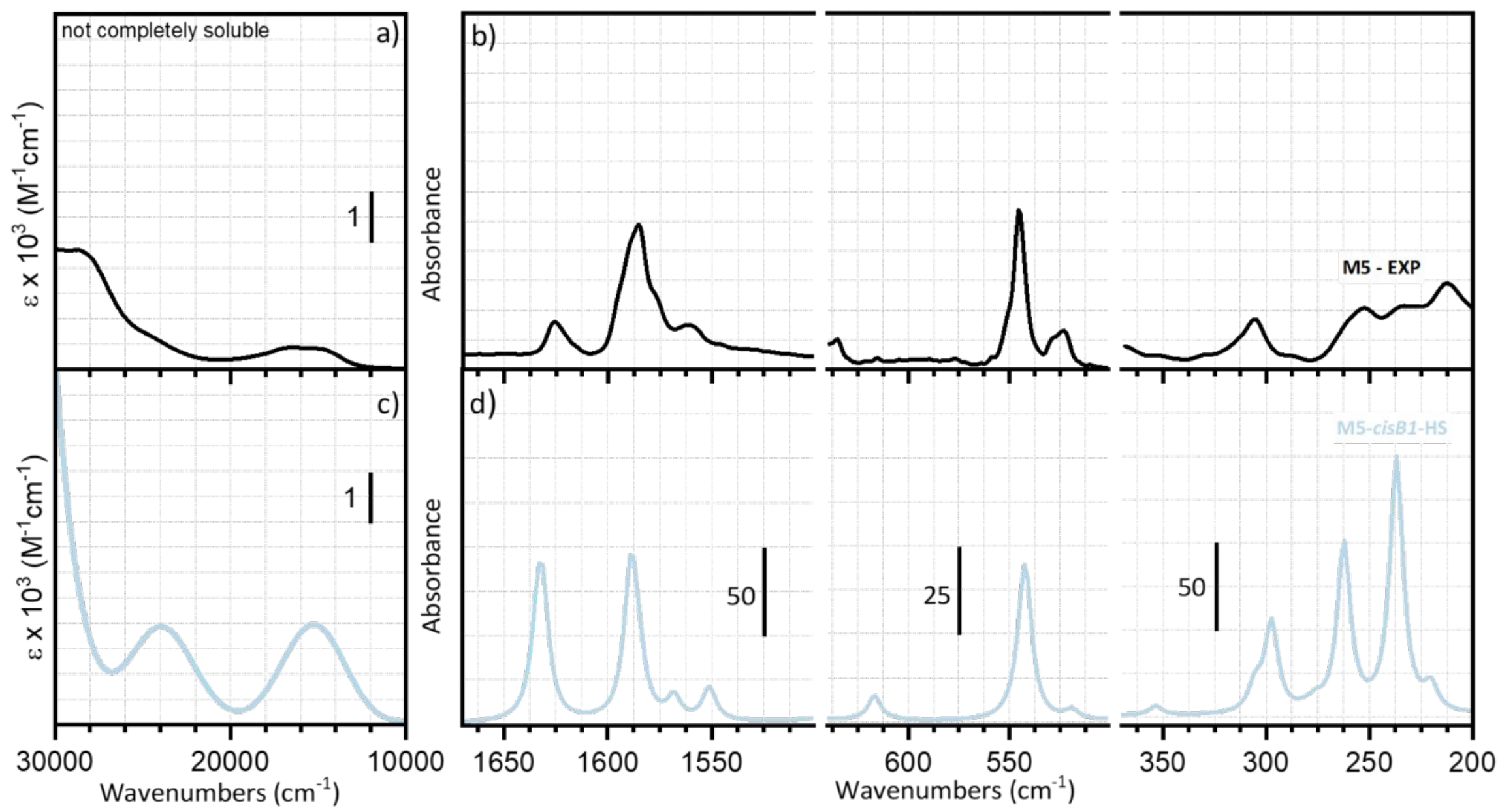

Figure S7. Comparison between experimental and simulated (a,c) UV-Vis and (b,d) IR spectra for **M5**, considering the **M5-*cisB1*-HS** isomer. The simulated UV-Vis spectra have been uniformly shifted by 5000 cm^{-1}.
